# MiR-200c-3p targets SESN1 and represses the IL-6/AKT loop to prevent cholangiocyte activation and cholestatic liver fibrosis

**DOI:** 10.1038/s41374-021-00710-6

**Published:** 2021-12-08

**Authors:** Yongfeng Song, Melanie Tran, Li Wang, Dong-Ju Shin, Jianguo Wu

**Affiliations:** 1 Department of Physiology and Neurobiology, University of Connecticut, Storrs, CT USA; 2 Department of Endocrinology and Metabolism, Shandong Provincial Hospital affiliated to Shandong First Medical University, Shandong Institute of Endocrinology & Metabolism, Shandong, China; 3 Independent Researcher, Tucson, AZ USA; 4 Department of Inflammation and Immunity, Lerner Research Institute, Cleveland Clinic, Cleveland, OH USA; 5 Department of Molecular Medicine, Cleveland Clinic Lerner College of Medicine of Case Western Reserve University, Cleveland, OH USA

**Keywords:** Mechanisms of disease, Experimental models of disease

## Abstract

Cholestasis causes ductular reaction in the liver where the reactive cholangiocytes not only proliferate but also gain a neuroendocrine-like phenotype, leading to inflammatory cell infiltration and extracellular matrix deposition and contributing to the development and progression of cholestatic liver fibrosis. This study aims to elucidate the role of miR-200c in cholestasis-induced biliary liver fibrosis and cholangiocyte activation. We found that miR-200c was extremely abundant in cholangiocytes but was reduced by cholestasis in a bile duct ligation (BDL) mouse model; miR-200c was also decreased by bile acids in vitro. Phenotypically, loss of miR-200c exacerbated cholestatic liver injury, including periductular fibrosis, intrahepatic inflammation, and biliary hyperplasia in both the BDL model and the 3,5-diethoxycarbonyl-1,4-dihydrocollidine (DDC) model. We identified sestrin 1 (SESN1) as a target of miR-200c. *Sesn1*^−/−^-BDL mice showed mitigation of cholestatic liver injury. On a molecular level, the pro-proliferative IL-6/AKT feedback loop was activated in *Mir200c*^−/−^ livers but was inhibited in *Sesn1*^−/−^ livers upon cholestasis in mice. Furthermore, rescuing expression of miR-200c by the adeno-associated virus serotype 8 ameliorated BDL-induced liver injury in *Mir200c*^−/−^ mice. Taken together, this study demonstrates that miR-200c restrains the proliferative and neuroendocrine-like activation of cholangiocytes by targeting SESN1 and inhibiting the IL-6/AKT feedback loop to protect against cholestatic liver fibrosis. Our findings provide mechanistic insights regarding biliary liver fibrosis, which may help to reveal novel therapeutic targets for the treatment of cholestatic liver injury and liver fibrosis.

## Abstract (web summary)

Cholangiocytes are the primary targets of cholangiopathies. This study elucidates the role of miR-200c in maintaining cholangiocyte homeostasis making use of cell culture and mouse models of cholestasis. MiR-200c restrains the proliferative and neuroendocrine-like activation of cholangiocytes by targeting sestrin 1(SESN1) and inhibiting the IL-6/AKT feedback loop to prevent cholestatic liver injury. The findings provide critical mechanistic insights into biliary liver fibrosis and suggest miR-200c may be a novel therapeutic target of cholangiopathies.

## Introduction

Cholestatic liver diseases arise from intrahepatic retention of cytotoxic bile acids (BAs) because of impaired bile formation and/or flow, accounting for a significant part of end-stage liver disease. Structural blockage, toxic substances, immunologic dysregulation, and congenital defects can be the etiology. BA-contributed injury to bile ducts and hepatocytes can lead to abnormal liver biochemistry, ductular reaction, biliary fibrosis, and disease progression to cirrhosis^1^. The activation and transdifferentiation of quiescent hepatic stellate cells (HSCs) into fibrogenic myofibroblasts are the key cellular events in liver fibrosis. HSCs increasingly express ACTA2 (actin alpha 2, smooth muscle; alias α-SMA) and other proteins (*e.g*., collagens) forming the connective tissue and synthesize and release tissue inhibitors of metalloproteinase (TIMP)-1 and -2. Despite these, a recognized common notion is that liver fibrosis is also impacted by other liver cell types and regulated by a broad spectrum of cytokines, chemokines, growth factors, and hormones^2^.

Cholangiopathies, such as primary biliary cholangitis (PBC) and primary sclerosing cholangitis (PSC), are characterized by biliary fibrosis and cholangiocytes as their primary targets. Cholestasis undermines the normal function of biliary epithelia and activates cholangiocytes. Reactive cholangiocytes proliferate and gain a neuroendocrine-like phenotype, constituting a cholangio-compartment promoting ductular reaction and periductular fibrosis. Cholangiocytes play an essential role not only in sustaining biliary proliferation itself but also in immune responses and hepatic inflammation. Reactive cholangiocytes secrete a range of different proinflammatory cytokines and chemokines (e.g., IL-6), which mediate the crosstalk with other hepatic cell types^3^.

IL-6 is an inflammatory cytokine and involved in cholangiocyte proliferation^4^. Cholangiocytes not only express receptors but also release mediators influencing cell proliferation, *e.g*., IL-6, thus constituting an autocrine feedback loop to amplify the proliferative response in ductular reaction^5,6^. In cholangiocytes, IL-6 activates the MAPK (mitogen-activated protein kinase and the PI3K (phosphatidylinositol-3 kinase)/AKT pathways to promote proliferation^7^. A reciprocal activation between IL-6 and AKT has been noticed. IL-6 elicits AKT activation^8^, while AKT activation promotes IL-6 production^9–11^.

MicroRNAs (miRNAs) are a class of small and regulatory non-coding RNAs (ncRNAs) of 21–25 nucleotides in length and control gene expression by degrading target mRNAs or suppressing protein translation^12^. Over one-third of human genes are targets of conserved miRNAs, which are involved in various biological processes. Recent studies have discovered several miRNAs that are involved in the progression of cholestatic liver fibrosis^13^. The miR-200 family comprises 5 members: miR-200a, miR-200b, miR-200c, miR-141, and miR-429. Expression levels of miR-200a and miR-200b were increased during liver fibrosis in mice^14^ and in fatty liver disease in rats^15^. Moreover, upregulation of miR-200b stimulated proliferation and migration of HSCs via the PI3K/AKT signaling pathway^16^, whereas upregulation of miR-200a inhibited TGF-*β*1-induced HSCs activation and proliferation^17^, suggesting the functional complexity of miR-200 family. Despite these advances, limited information is available regarding the specific function of miR-200 family in cholangiocytes and cholestatic liver injury.

Sestrin 1 (SESN1), also known as PA26, is a member of the stress-responsive gene family. SESN1 is a p53-target gene and confers resistance to p53-induced oxidative stress through facilitating regeneration of peroxiredoxins^18^. Independently of their redox activity, SESN1 inhibits expression of mTOR, thus mediating its aging-related function^19^. However, the pathophysiological function of SESN1 in the liver remains undetermined.

This study aims at understanding the function of miR-200c in cholestatic liver fibrosis making use of gene knockout mice. We identified SESN1 as a novel target of miR-200c. We elucidated a new miR-200c/SESN1 regulatory axis contributing to IL-6/AKT-mediated cholangiocyte activation in the development of cholestatic liver fibrosis.

## Materials and methods

### Animals

The whole body *Mir200c*^−/−^ mice on a mixed 129/C57 background were recently generated^20^. MiR-141, clustered with miR-200c, was also deleted due to technical difficulties when generating the strain. *Sesn1*^−/−^ mice (C57BL/6 N) were purchased from the Jackson Laboratory (Stock No. 027566). Mice were fed a standard rodent chow diet (Harlan, No. 2018) with free access to water and housed in a temperature-controlled (23 °C), pathogen-free room with a 12-h light and 12-h dark cycle. In vivo experiments were performed on male mice at the age of 6 weeks unless stated otherwise (n **=** 5–10 mice/group). The treatment of mice with 3,5-diethoxycarbonyl-1,4-dihydrocollidine (DDC)-supplemented diet and the bile duct ligation (BDL) surgery have been described previously^21,22^. For in vivo viral transduction, mice were injected via tail vein with the purified adeno-associated virus serotype 8 (AAV8) containing a liver-specific thyroxine-binding globulin (TBG) promoter driving miR-200c expression at 5 × 10^10^ virus particles per mouse^23^. All mice were sacrificed after overnight fasting unless otherwise indicated. Whole blood was collected, and serum was obtained by centrifugation (1,500 rpm, 15 min) and stored at −80 °C until analysis. Basic procedures to analyze serum biochemistry parameters, including alanine aminotransferase (ALT), aspartate aminotransferase (AST), total bilirubin (TBIL), and direct bilirubin (DBIL), were described previously^24^. The serum BA concentrations were measured with a Colorimetric Total Bile Acid Assay Kit (#STA-631, CELL BIOLABS, Inc). H&E, Masson Trichrome, Sirius Red, and immunohistochemistry (IHC) staining were performed. Five fields of view per slide per mouse liver were randomly taken under a microscope and quantified by ImageJ. The sample size is the number of mice in each experimental group, which was shown in figure legends and Supporting Information. Protocols for animal use were approved by the IACUC at the University of Connecticut.

### Isolation of primary mouse hepatocytes, HSCs, Kupffer cells (KCs), and cholangiocytes

Primary mouse hepatocytes were isolated using a standard two-step collagenase digestion method as previously described^25^. Primary HSCs and KCs were isolated using Percoll gradient centrifugation method as previously described^26^. For cholangiocytes, the biliary tree was digested with collagenase, hyaluronidase, and bovine pancreas trypsin, filtered in a 70 μm cell strainer, and then purified by immunoaffinity separation as previously described^27^.

### Other standard methods

Luciferase assays, mutagenesis, qPCR, Western blot (WB), and IHC were described previously^24,28^. For WB, equal amounts of protein from individual mouse liver in each group (*n* **=** 5–10/group) were pooled, and single or duplicate loadings were used. The bands were quantified using ImageJ and the relative expression was denoted. Primers and additional materials and methods were detailed in Supporting Information.

### Statistical analysis

Data are shown as the mean ± standard error of the mean (SEM) and are representative of at least three independent experiments. Normal distribution of the data was examined using the Shapiro-Wilk test and data was log-transformed as necessary to obtain a normal distribution. Statistical analysis was carried out using the Student’s *t*-test between two groups and one-way ANOVA followed by Tukey’s post hoc test for multiple groups. *P* < 0.05 was considered statistically significant.

## Results

### BAs enhance cholangiocyte activation by diminishing expression of miR-200c

**Fig. 1:**
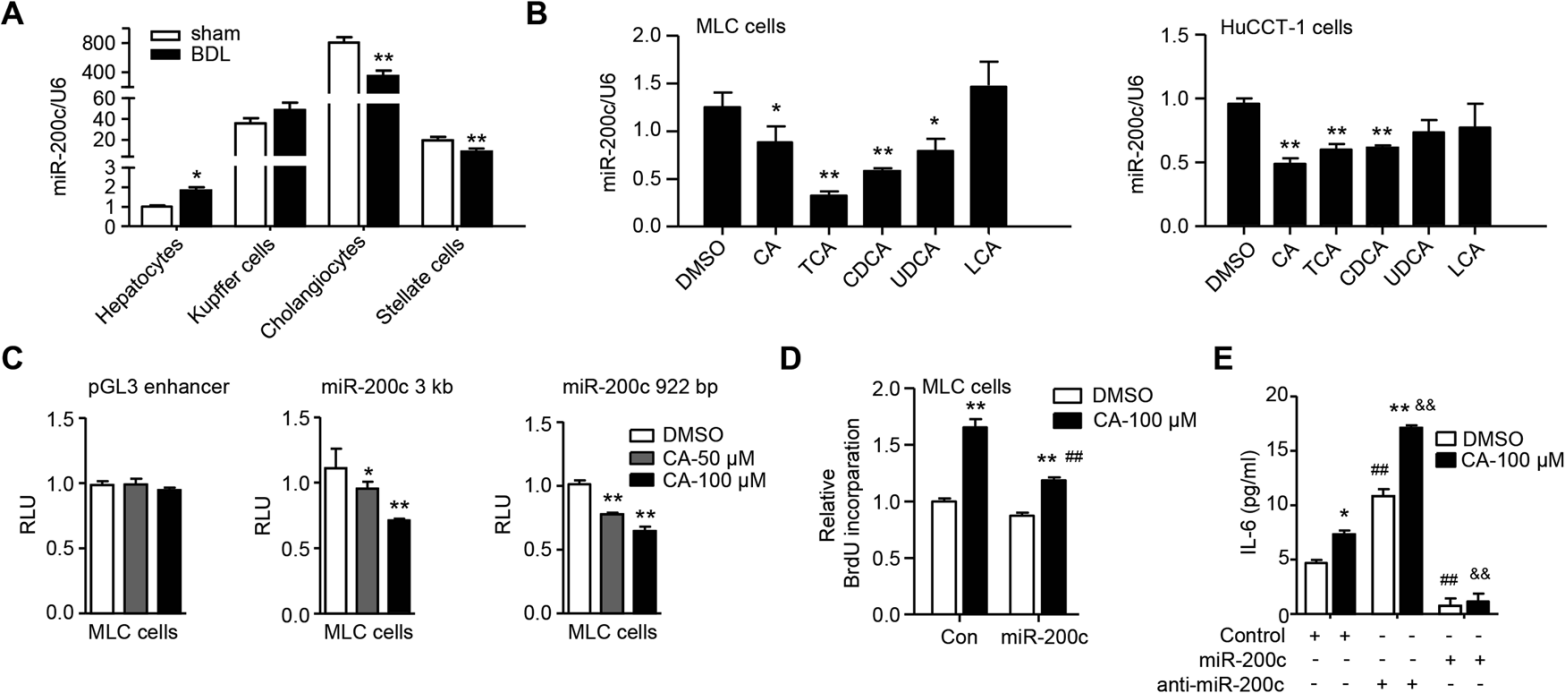
BAs decrease expression of miR-200c to promote IL-6-mediated cholangiocyte proliferation. **A** qPCR of the expression level of miR-200c in primary cells isolated from WT mouse livers 1 week after sham or BDL surgery. Data are shown as mean ± SEM (*n* = 5/group). *P < 0.05 & **P < 0.01 vs. sham. **B** qPCR of the expression level of miR-200c in mouse large cholangiocytes (MLC) and human cholangiocarcinoma cells (HuCCT-1). MLC and HuCCT-1 cells were treated with CA (100 µM), TCA (100 µM), CDCA (100 µM), UDCA (100 µM), or LCA (10 µM) for 24 h. Data are shown as mean ± SEM. *P < 0.05 & **P < 0.01 vs. DMSO. **C** Transient transfection to determine luciferase reporter activity of miR-200c promoter regulated by CA. Data are shown as mean ± SEM. *P < 0.05 & **P < 0.01 vs. DMSO. **D** Cell proliferation was determined by BrdU incorporation. MLC cells were transfected with control (con) or miR-200c plasmid for 24 h, followed by the treatment with DMSO or CA for 48 h in the presence of BrdU. Data are shown as mean ± SEM. **P < 0.01 vs. DMSO, ^##^*P* < 0.01 vs. con-CA. **E** ELISA of the expression level of IL-6. MLC cells were transfected with control miRNA, miR-200c, or miR-200c inhibitor (anti-miR-200c), and then treated with DMSO or CA (100 µM) for 48 h. The secreted form of IL-6 was determined by ELISA. Data are shown as mean ± SEM. *P < 0.05 & **P < 0.01 CA vs. DMSO; ^##^*P* < 0.01 vs. control-DMSO; ^&&^*P* < 0.01 vs. control-CA.

Among the primary mouse hepatic cells, cholangiocytes expressed 20 to 40 folds higher levels of miR-200c than HSCs and KCs. The expression level of miR-200c in cholangiocytes was about 800 folds higher than that in hepatocytes, implying its potential role in maintaining biliary homeostasis (Supporting Fig. 1A). The expression level of miR-200c was decreased by about 50% in cholangiocytes 1 week after BDL surgery. On the contrary, it was increased in hepatocytes by BDL (Fig. 1A and Supporting Fig. 1B).

BDL results in BA accumulation in the liver, disrupts epithelial tight junctions and barrier integrity of bile ducts, and leads to direct infiltration of hepatic cells by BAs. The BA composition and dynamics in BDL-mice have been elucidated before^29^. Although some secondary BAs, *e.g*., taurochenodeoxycholic acid (TDCA) and lithocholic acid (LCA), show decreasing trends, most of the hepatic BAs increase as early as 6 h post-BDL and then subside in BDL-mice^29^. BAs in livers of BDL-mice gradually become more hydrophilic (from hydrophilic to hydrophobic: muricholic acid (MCA) > ursodeoxycholic acid (UDCA) > cholic acid (CA) > chenodeoxycholic acid (CDCA) > deoxycholic acid (DCA) > LCA; taurine-conjugated > glycine-conjugated > unconjugated)^30–32^. Rather than cause direct toxicity, BAs may act as inflammagens after BDL in mice^29^. Numerous in vitro studies have investigated the effect of BAs on different liver cell types^27,33,34^. How BAs regulate expression of miR-200c remains undetermined. We examined the expression level of miR-200c in cholangiocytes exposed to several BAs, which represent distinct hydrophilicity and are the major BA species in both mouse and human BA pools, although the level of LCA is very low in mice^30^. As the expression level of miR-200c was much higher in MLC and human HuCCT-1 than MSC cells (Supporting Fig. 1C), we used MLC and HuCCT-1 for further studies. Expression of miR-200c was reduced in both MLC and HuCCT-1 cells treated with several BAs, including CA, taurocholic acid (TCA), CDCA and/or UDCA, for 24 h and further decreased in MLC cells exposed to CA for 48 h (Fig. 1B and Supporting Fig. 1D). Luciferase assays using reporters containing upstream sequences (−3 kb and −922 bp) of human pri-miR-200c demonstrated that the reporter activity was dose-dependently inhibited by CA (Fig. 1C), suggesting a suppressive effect of CA on the transcription of miR-200c. We predicted the transcription factors (TFs) that potentially bind miR-200c promoter (the above −922 bp sequence) using the online tool ALGGEN-PROMO. In addition to Sp1 known to activate transcription of miR-200c^35^, among the many predicted TFs, HNF1A’s binding site has a significant score and is conserved in mice and humans. CA impaired the activation of miR-200c promoter by Sp1, while promoted the inhibition of miR-200c promoter by HNF1A, suggesting CA might non-specifically affect the interaction between TFs and miR-200c promoter (Supporting Fig. 1E). Although TCA increases the level of S1PR2 mRNA in MLC cells^27^, we did not find significant changes of expression of mRNAs of BA receptors, including TGR5, S1PR2, VDR, FXR, and PXR, in MLC cells treated with CA (data not shown).

The proliferative and neuroendocrine-like activation of cholangiocytes contributes to ductular reaction in cholestatic liver disease. To determine whether miR-200c regulates cholangiocyte proliferation, MLC cells were transfected with miR-200c-expressing plasmids and then treated with CA. CA-treatment increased the proliferation of MLC cells as measured by BrdU incorporation, which was ameliorated by miR-200c overexpression (Fig. 1D), indicating that miR-200c confines the proliferation potential of cholangiocytes. CA-treatment also increased expression of the pro-proliferative IL-6 in MLC cells, which was augmented by the miR-200c inhibitor (anti-miR-200c) but prevented by miR-200c overexpression (Fig. 1E and Supporting Fig. 1F). A similar role of miR-200c in regulation of IL-6 expression was found in TCA-treated MLC cells (Supporting Fig. 1G). Taken together, these results suggest that cholangiocyte proliferation induced by BA-exposure is at least contributed by inhibition of miR-200c expression and activation of IL-6 expression.

### Loss of miR-200c aggravates the development of biliary fibrosis in mice

**Fig. 2:**
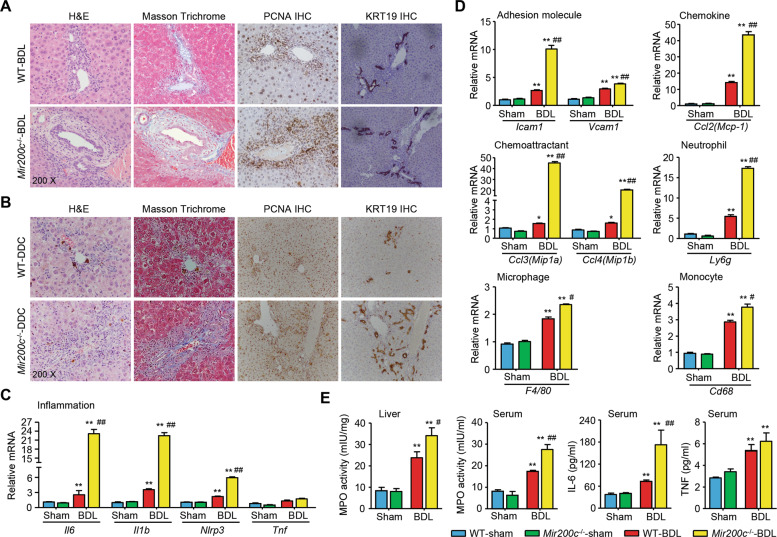
miR-200c-deficiency exacerbates cholestatic liver fibrosis in mice. **A**, **B** Representative images of H&E, Masson Trichrome, and IHC of PCNA and KRT19 staining of liver sections in BDL (**A**) and DDC (**B**) models. WT and *Mir200c*^−/−^ mice were subjected to sham or BDL for 1 week (*n* = 10 mice/group) or fed a chow or DDC diet for one month (*n* = 7 mice/group). **C**, **D** qPCR of gene expression in livers from sham- or BDL-operated mice. Data are shown as mean ± SEM (*n* = 10 mice/group). *P < 0.05 & **P < 0.01 vs. WT-sham; ^#^*P* < 0.05 & ^##^*P* < 0.01 vs. WT-BDL. **E** Measurement of liver and serum MPO activity, as well as serum levels of IL-6 and TNF. Data are shown as mean ± SEM (*n* = 10 mice/group). **P < 0.01 vs. WT-sham; ^#^*P* < 0.05 & ^##^*P* < 0.01 vs. WT-BDL.

BDL leads to ductal proliferative responses and rapid establishment of periportal fibrosis. Body and liver weights were similar between wild type (WT) and *Mir200c*^−/−^ mice before and after surgery (Supporting Fig. 2A, B). H&E, Masson Trichrome, and IHC of PCNA and KRT19 staining, did not identify differences in the liver between WT-sham and *Mir200c*^−/−^-sham mice (Supporting Fig. 3A). The levels of ALT, AST, DBIL, and BAs were comparably elevated in the serum of WT-BDL and *Mir200c*^−/−^-BDL mice compared to sham groups, although the BA level was lower in *Mir200c*^−/−^-BDL vs. WT-BDL mice (Supporting Fig. 3B). As expected, biliary fibrosis was aggravated in *Mir200c*^−/−^-BDL vs*.* WT-BDL mice, as revealed by H&E, Masson Trichrome (Fig. 2A and Supporting Fig. 4A), and Sirius Red staining (Supporting Fig. 4B). Bile infarct areas and PCNA- and KRT19-positive cells were also markedly increased in *Mir200c*^−/−^-BDL vs. WT-BDL mice (Fig. 2A and Supporting Fig. 4A).

DDC-feeding results in a reactive phenotype of cholangiocytes, renders bile duct injury, and induces biliary type liver fibrosis. Body and liver weights, liver histology, and hepatic expression of PCNA and KRT19 remained comparable between WT-chow and *Mir200c*^−/−^-chow mice; DDC-feeding similarly increased liver weight and liver/body weight ratio in both genotypes (Supporting Figs. 5 and 6A). The serum levels of AST, ALT, and DBIL showed similar increases between WT-DDC and *Mir200c*^−/−^-DDC mice; however, DDC-feeding reduced the serum BA level in *Mir200c*^−/−^ mice (Supporting Fig. 6B). Consistent with the BDL model, biliary proliferation and periductular fibrosis were aggravated in *Mir200c*^−/−^-DDC vs. WT-DDC mice, as indicated by the IHC staining of PCNA and KRT19, and the Masson Trichrome/Sirius Red staining, respectively (Fig. 2B and Supporting Fig. 7A and B). The severity of intrahepatic bile infarct was also increased in *Mir200c*^−/−^-DDC vs. WT-DDC mice (Supporting Fig. 7A).

Because the phenotypic changes were consistent in *Mir200c*^−/−^-BDL and *Mir200c*^−/−^-DDC mice, subsequent studies were focused on the BDL model. Loss of miR-200c increased expression of multiple genes related to inflammation, including *Il6*, *Il1b*, *Nlrp3*, and *Tnf*) in BDL mice (Fig. 2C). The expression levels of adhesion molecules (*Icam1*, *Vcam1*), a chemokine (*Ccl2*, alias *Mcp-1*), a neutrophil marker (*Ly6g*), chemoattractants for monocytes and/or neutrophils (*Ccl3*, alias *Mip1a*; *Ccl4*, alias *Mip1b*), a macrophage marker (*Adgre1*, alias *F4/80*), and a monocyte linage marker (*Cd68*) were analyzed. Except *Tnf*, hepatic expression levels of these genes were all induced by BDL compared to sham, which was further increased by miR-200c-deficiency (Fig. 2D). Similarly, liver and serum myeloperoxidase (MPO) activity, as a marker of neutrophil infiltration^36^, and serum IL-6 levels were further elevated in *Mir200c*^−/−^-BDL vs. WT-BDL mice. On the other hand, the level of serum TNF was similarly induced in *Mir200c*^−/−^-BDL and WT-BDL mice (Fig. 2E). Thus, these results suggest that miR-200c-deficiency exacerbated cholestatic liver fibrosis by activating genes associated with cholangiocyte proliferation, fibrogenesis, and inflammation.

### SESN1 functions downstream of miR-200c to regulate IL-6-mediated cholangiocyte proliferation

**Fig. 3:**
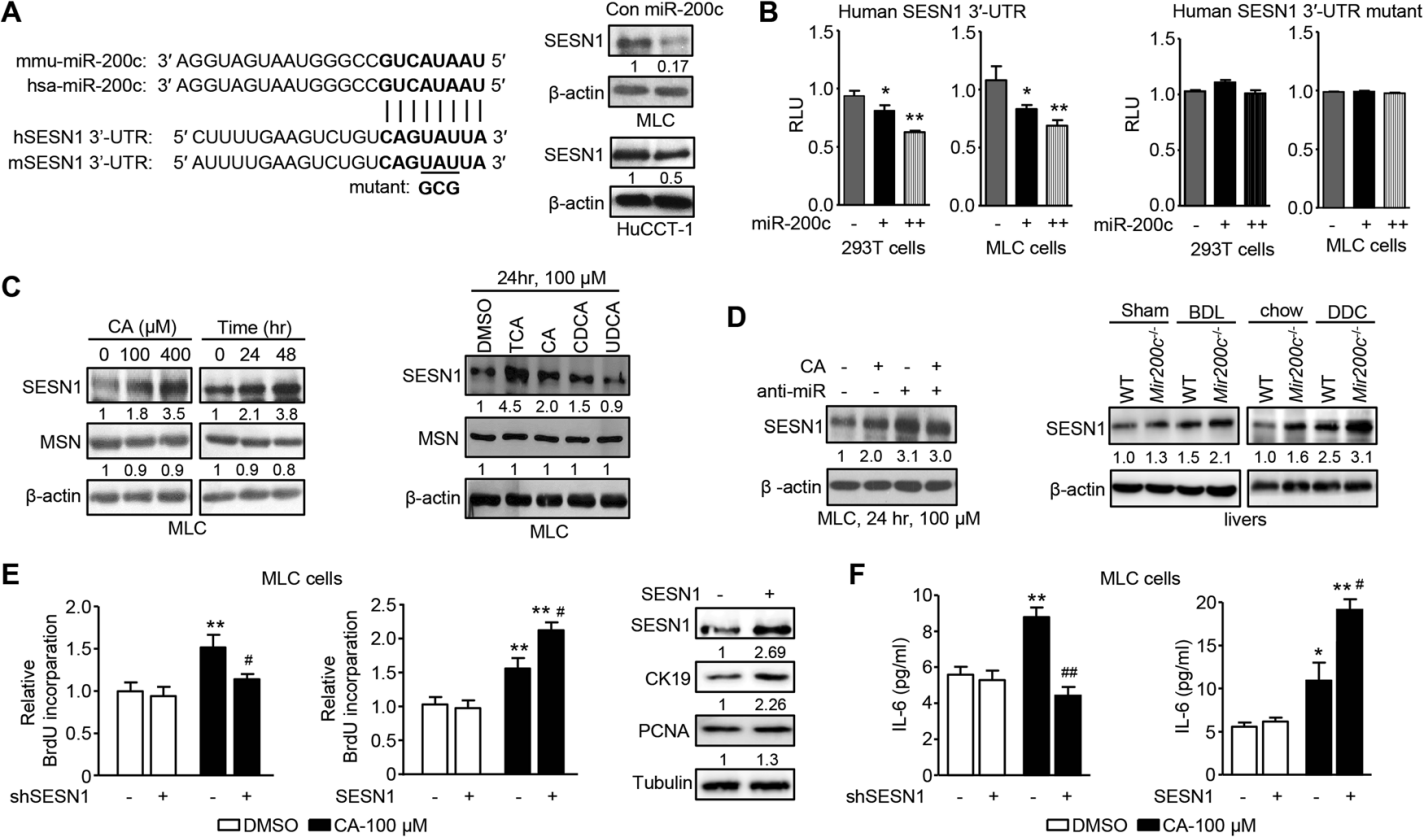
Identification of SESN1 as a miR-200c target gene. **A** Left: Diagram showing the binding sites of miR-200c in the 3’-UTR of SESN1 mRNA in humans (hSESN1) and mice (mSESN1). Right: WB of the expression level of SESN1. MLC and HuCCT-1 cells were transfected with control (con) or miR-200c plasmid for 48 h and total proteins were isolated for the assay. **B** Left: Transient transfection to determine luciferase reporter activity of SESN1 3’-UTR regulated by miR-200c. Right: Mutagenesis assays. A mutant luciferase reporter construct of SESN1 3’-UTR, as shown in **A**, was generated and used for transient transfection. Data are shown as mean ± SEM. *P < 0.05 & **P < 0.01 vs. control miRNA (−). **C** WB of indicated proteins in MLC cells treated with CA of different concentrations for 24 h (left), CA (100 µM) for different time (left), or different BAs (100 µM) for 24 h (right). **D** Left: WB of SESN1 protein in MLC cells transfected with the miR-200c inhibitor (anti-miR) in the presence or absence of CA treatment. Right: WB of SESN1 protein in WT and *Mir200c*^−/−^ livers from the BDL or DDC model. **E** BrdU incorporation to determine cell proliferation. Left: MLC cells were transfected with control (−) or shSESN1 (+) shRNA for 24 h, followed by the treatment with DMSO or CA for 48 h in the presence of BrdU. Middle: MLC cells were transfected with the empty vector (−) or SESN1 plasmid (+) for 24 h. Then, the cells were treated with DMSO or CA for 48 h in the presence of BrdU. Right: WB of indicated proteins in MLC cells after SESN1 overexpression. Data are shown as mean ± SEM. **P < 0.01 vs. DMSO; ^#^*P* < 0.05 & ^##^*P* < 0.01 vs. (−)-CA. **F** ELISA of IL-6 secretion. Left: MLC cells were treated as in (**E**, left). Right: MLC cells were treated as in (**E**, middle). Data are shown as mean ± SEM. *P < 0.05 & **P < 0.01 vs. DMSO; ^#^*P* < 0.05 & ^##^*P* < 0.01 vs. (−)-CA.

To better understand the underlying mechanisms by which miR-200c protects against cholestatic liver fibrosis, it is critical to identify new miR-200c target genes. We used multiple online prediction tools (TargetScan, miRDB, PicTar, RNA22, and miRanda) to increase the stringency. A conserved seed match region of miR-200c was found in the 3’-UTR of SESN1 mRNA in both humans (hSESN1) and mice (mSESN1) (Fig. 3A left). Based on the binding free energy, the probability of miR-200c binding to the 3’-UTR of SESN1 mRNA in humans (−19.7 kcal/mol) and mice (−23.9 kcal/mol) was 98 and 95%, respectively, indicating a high likelihood of binding (Supporting Figs. 8 and 9). As expected, miR-200c overexpression reduced expression of SESN1 at both protein and mRNA levels in MLC cells and HuCCT-1 cells (Fig. 3A right and Supporting Fig. 10A). We constructed a luciferase reporter containing hSESN1 3’-UTR region for further validation (Supporting Fig. 10B). MiR-200c repressed the activity of hSESN1 3’-UTR reporter in a dose-dependent manner in 293 T and MLC cells (Fig. 3B left), whereas the effect of miR-200c was abolished when its binding site was mutated (Fig. 3B right). In contrast, miR-200c did not affect mRNA levels of other predicted targets, such as Moesin (*Msn*) (Supporting Fig. 10C).

As CA decreased the expression level of miR-200c (Fig. 1B), we asked whether it could induce SESN1 expression. Indeed, western blot showed that CA increased expression of SESN1 in a dose- and time-dependent manner but had no effect on MSN (Fig. 3C left). Among the several BAs, TCA exhibited the highest potency to induce expression of SESN1 (Fig. 3C right). CA induced SESN1 expression but failed to increase its expression in the presence of the miR-200c inhibitor (anti-miR-200c), suggesting that CA-induction of SESN1 expression was mediated at least through miR-200c (Fig. 3D left). Hepatic expression of SESN1 was induced by BDL and DDC in both WT and *Mir200c*^−/−^ mice. Also, miR-200c-deficiency potentiated hepatic expression of SESN1 in both sham- and BDL-operated mice, as well as in both chow- and DDC-fed mice (Fig. 3D right). These results were consistent with our in vitro results demonstrating that SESN1 was a direct target gene of miR-200c.

We interrogated whether SESN1 promoted cholangiocyte proliferation and IL-6 production. In the presence of CA, knockdown of SESN1 (Supporting Fig. 10D) inhibited BrdU incorporation (Fig. 3E left) and IL-6 production (Fig. 3F left), while overexpression of SESN1 potentiated CA-induced BrdU incorporation (Fig. 3E middle) and IL-6 production (Fig. 3F right). Further, SESN1 overexpression increased expression of KRT19 (Fig. 3E right). These results suggested that SESN1 facilitated IL-6-mediated cholangiocyte proliferation.

### *Sesn1*-deficiency prevents the development of cholestatic liver fibrosis in mice

**Fig. 4:**
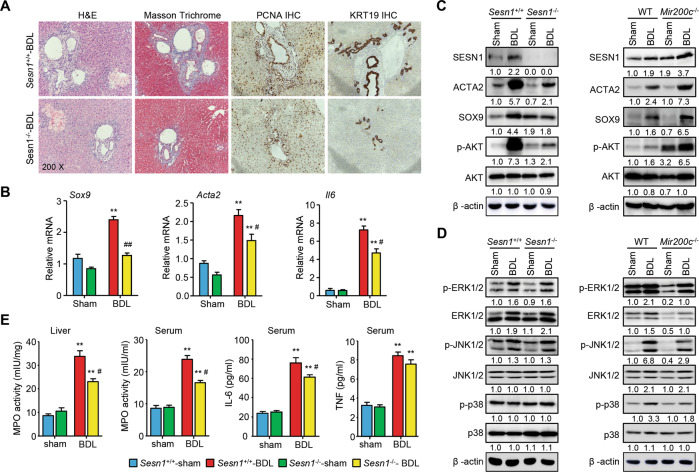
*Sesn1*-deficiency alleviates cholestatic liver fibrosis. A Representative images of H&E, Masson Trichrome, and IHC of PCNA and KRT19 staining of liver sections in *Sesn1*^+/+^ and *Sesn1*^−/−^ mice subjected to BDL for 1 week (*n* = 10 mice/group). **B** qPCR of gene expression in livers from *Sesn1*^+/+^ and *Sesn1*^−/−^ mice subjected to sham or BDL for 1 week. **C**, **D** WB of proteins in the livers from the indicated mice 1 week post-sham or -BDL surgery. **E** MPO activity in the liver and serum and levels of IL-6 and TNF in the serum from mice in **B**. All data are shown as mean ± SEM (*n* = 10 mice/group). **P < 0.01 vs. *Sesn1*^+/+^-sham; ^#^*P* < 0.05 & ^#*#*^*P* < 0.01 vs. *Sesn1*^+/+−^BDL.

No significant changes were observed in liver histology, biliary proliferation, and liver fibrosis in *Sesn1*^−/−^-sham vs*. Sesn1*^+/+^-sham mice (Supporting Fig. 11A). Serum levels of ALT, AST, bilirubin, and BAs did not show a significant reduction in *Sesn1*^−/−^-BDL vs*. Sesn1*^+/+^-BDL mice (Supporting Fig. 11B). H&E, Masson Trichrome, and Sirius Red staining revealed a marked reduction in periductular fibrosis in *Sesn1*^−/−^-BDL vs*. Sesn1*^+/+^-BDL mice; intrahepatic proliferation and biliary hyperplasia were also ameliorated, as revealed by IHC of PCNA and KRT19 (Fig. 4A and Supporting Fig. 12A, B). Consistently, hepatic expression levels of genes, including a progenitor cell marker (*Sox9*), a liver fibrosis marker (*Acta2*), and an inflammation marker (*Il6*), were decreased in *Sesn1*^−/−^-BDL vs*. Sesn1*^+/+^-BDL mice (Fig. 4B). In contrast, expression of many genes involved in BA metabolism, fibrosis, and inflammation remained at comparable levels between *Sesn1*^−/−^-BDL and *Sesn1*^+/+^-BDL mice (Supporting Fig. 13).

Corresponding to the induction of mRNAs, ACTA2 and SOX9 proteins in the liver were highly induced by BDL in *Sesn1*^+/+^ mice; however, the induction was diminished by *Sesn1*-deficiency (Fig. 4C left). Examination of several signaling pathways revealed a striking elevation of p-AKT protein in *Sesn1*^+/+^-BDL, which was greatly dampened in *Sesn1*^−/−^-BDL livers (Fig. 4C left and Fig. 4D left), suggesting that SESN1 contributed to AKT activation in cholestasis. Hepatic expression of SESN1, ACTA2, SOX9, and p-AKT proteins were induced by BDL, which was further potentiated by miR-200c deficiency (Fig. 3C right). On the other hand, the levels of p-ERK1/2, p-JNK1/2, and p-p38 proteins did not noticeably change in *Sesn1*^−/−^-BDL vs. *Sesn1*^+/+^-BDL livers (Fig. 4D left). In contrast to enhancement of p-AKT expression (Fig. 3C right), p-ERK1/2, p-JNK1/2, and p-p38 proteins in the liver were moderately reduced in *Mir200c*^−/−^-BDL vs. WT-BDL mice (Fig. 4D right). These results demonstrated that *Sesn1*-deficiency diminished AKT activation to protect against the intrahepatic proliferation and biliary hyperplasia in cholestasis. Moreover, MPO activities in the liver and serum were reduced in *Sesn1*^−/−^-BDL vs. *Sesn1*^+/+^-BDL mice (Fig. 4E). The serum level of IL-6 but not TNF was significantly attenuated by *Sesn1*-deficiency in BDL-operated mice (Fig. 4E). Therefore, the above results suggested that SESN1 contributed to AKT activation and IL-6 expression to regulate the development of cholestatic liver injury.

### Rescuing expression of miR-200c alleviates biliary fibrosis in *Mir200c*^−/−^-BDL mice

**Fig. 5:**
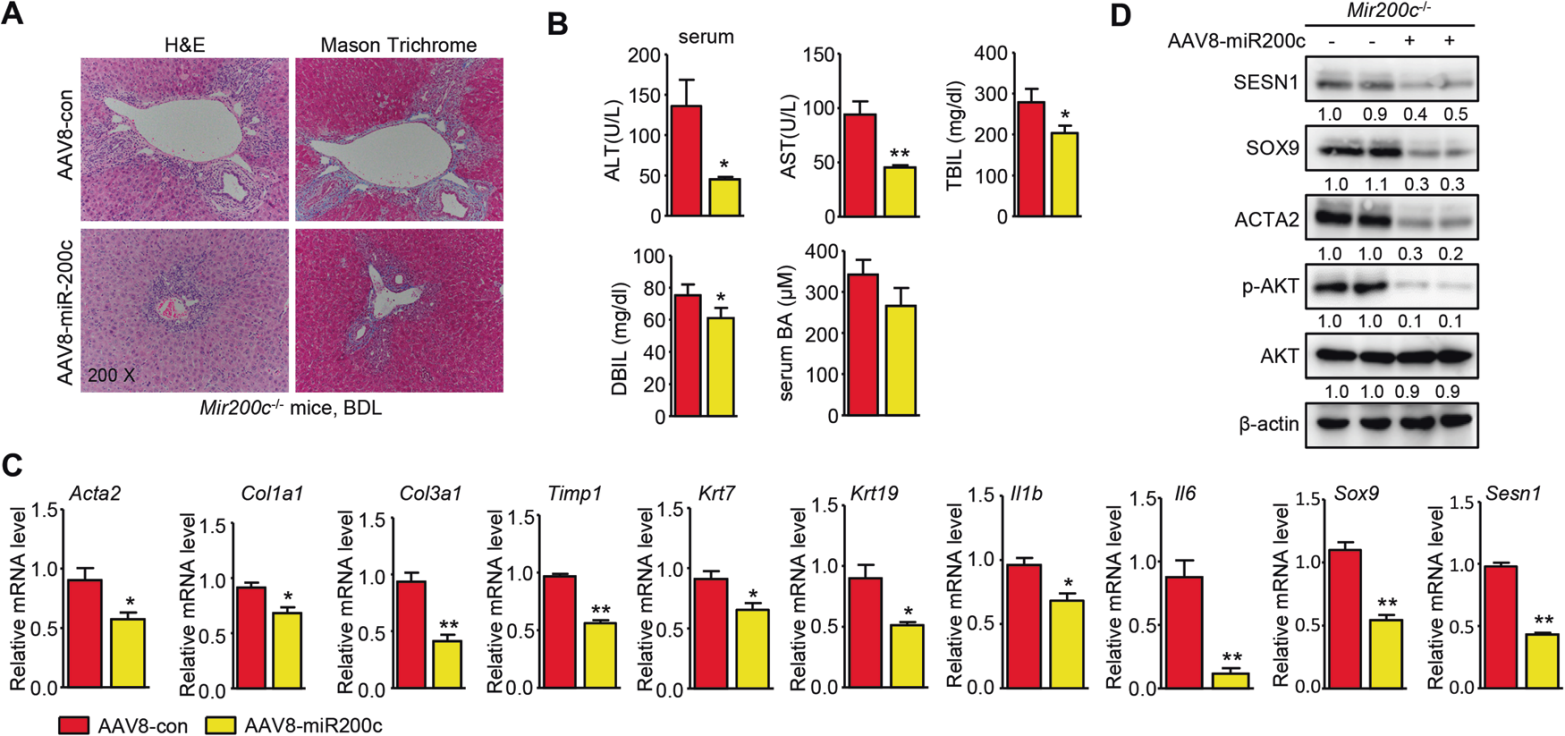
Reconstitution of miR-200c in *Mir200c*^−/−^ mice prevents the progression of cholestatic liver fibrosis. **A** Representative images of H&E and Masson Trichrome staining of liver sections. *Mir200c*^−/−^ mice were transduced with AAV8-con (control) or AAV8-miR-200c (overexpression) viruses via tail vein injection for one month, followed by sham or BDL for 1 week (*n* = 5 mice/group). **B**, **C**, and **D** Levels of ALT, AST, TBIL, DBIL, and BAs in the serum (**B**), as well as qPCR of gene expression (**C**) and WB of protein expression (**D**) in the livers of mice from (**A**). All data are shown as mean ± SEM (*n* = 5 mice/group). *P < 0.05 &**P < 0.01 vs. AAV8-con.

We conducted rescue experiments in *Mir200c*^−/−^ mice transduced with AAV8-miR-200c or AAV8- control (con) to further confirm the role of miR-200c in BDL-induced cholestatic liver fibrosis (Supporting Fig. 14A). H&E and Masson Trichrome staining showed remarkable attenuation of periportal fibrosis in miR-200c-BDL vs. con-BDL mice (Fig. 5A). The reduced liver injury was accompanied by decreased serum levels of ALT, AST, and bilirubin, while the serum level of BAs did not significantly change (Fig. 5B). We also checked hepatic mRNA expression of the major regulators of the BA biosynthesis pathways. Expression of *Cyp7a1* and *Cyp27a1*, the rate-limiting enzyme of BA synthesis in the classic pathway and the alternative pathway respectively, had no significant difference between miR-200c-BDL and con-BDL mice. Interestingly, gene expression of another BA synthesis enzyme, *Cyp8b1*, was reduced in miR-200c-BDL vs. con-BDL mice. Expression of *Bsep*, a gene for the hepatic canalicular bile salt export pump, was also moderately reduced in miR-200c-BDL mice. The mRNA levels of *Ntcp*, *Fxr*, and *Oatp1b2* had no significant changes; however, expression of *Shp*, a FXR target gene, was upregulated in miR-200c-BDL vs. con-BDL mice (Supporting Fig. 14B).

The mRNA levels of liver fibrosis markers (*Acta2*, *Col1a1*, *Col3a1*, and *Timp1*), cholangiocyte proliferation markers (*Krt7* and *Krt19*), inflammation markers (*Il6* and *Il1b*), and the progenitor cell marker (*Sox9*) were markedly decreased in miR-200c-BDL mice compared to con-BDL mice. Expression of *Sesn1* mRNA was also decreased in miR-200c-rescued mice (Fig. 5C). Expression of SESN1, ACTA2, SOX9, and p-AKT proteins were decreased in miR-200c-BDL mice vs. con-BDL mice (Fig. 5D). Taken together, these results demonstrated that miR-200c was protective in cholestatic liver fibrosis.

### Expression of miR-200c and SESN1 is dysregulated in human liver diseases

**Fig. 6:**
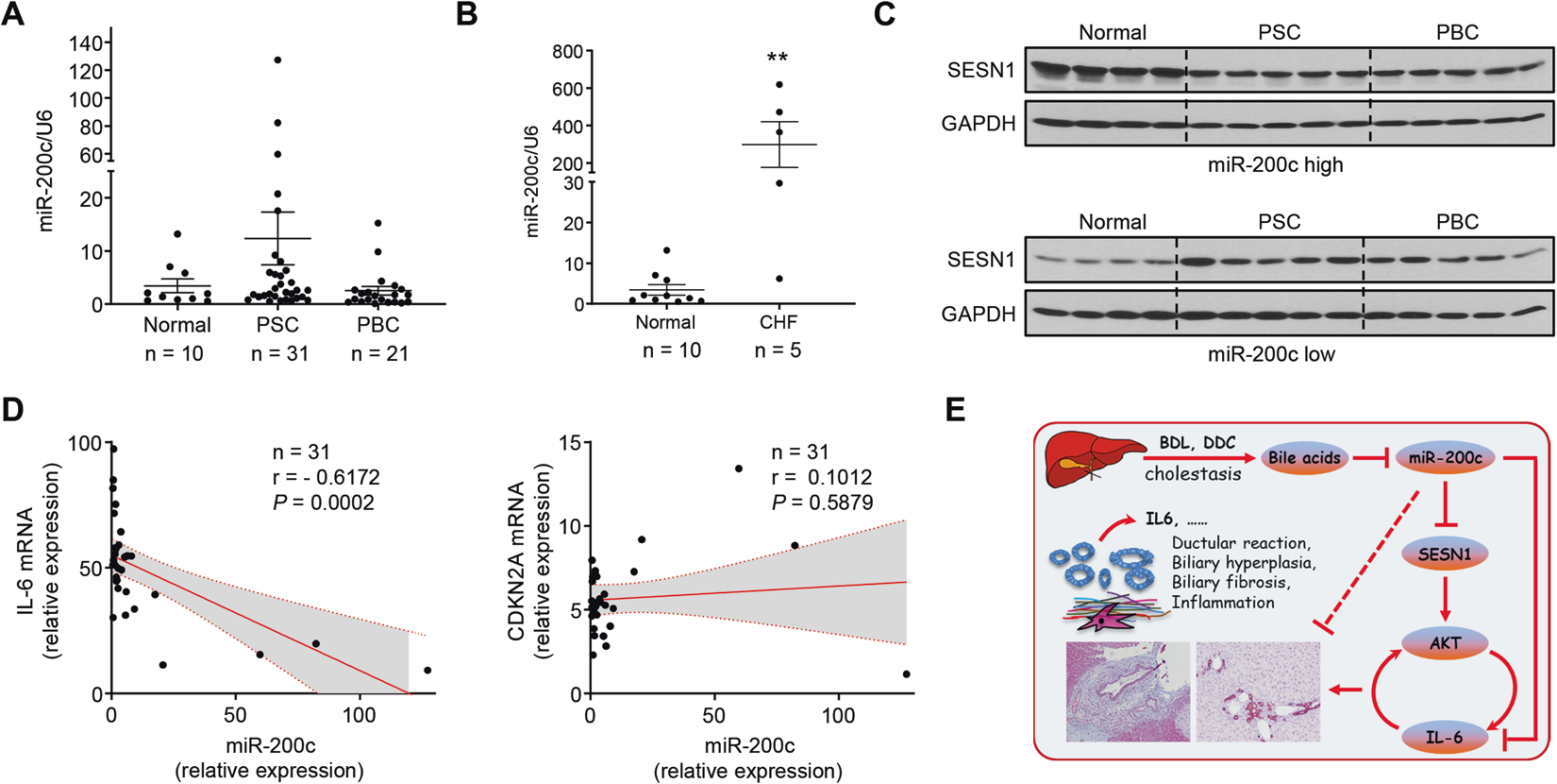
The expression level of SESN1 negatively correlates with that of miR-200c in human cholestatic liver diseases. **A**, **B** qPCR of miR-200c expression in human liver specimens. **P < 0.01 vs. normal controls. **C** Human liver specimens were divided into two groups, miR-200c high and miR-200c low, based on the expression level of miR-200c in disease livers vs. normal controls. Expression of SESN1 protein was determined by WB. **D** Pearson correlation of expression of miR-200c, IL-6, and CDKN2A mRNAs. Gene expression was determined by qPCR and normalized to normal controls. The relative fold change was used to plot the correlation. **E** A schematic showing that miR-200c inhibits cholestatic liver fibrosis via targeting SESN1 and inhibiting the IL-6/AKT feedback loop.

PSC and PBC, the most common immune-mediated chronic cholestatic liver diseases, are pathologically manifested by ductular reaction and periportal fibrosis. Despite the insignificance, the expression level of miR-200c seemed to be elevated in PSC but not PBC compared to normal livers (Fig. 6A). Congenital hepatic fibrosis (CHF) is a rare developmental disorder characterized by severe periportal fibrosis and proliferation of interlobular bile ducts. Expression of MiR-200c was significantly and remarkably increased in CHF livers (Fig. 6B). We divided the normal, PSC, and PBC liver specimens into two groups based on the relative expression of miR-200c vs. the normal controls: miR-200c high and miR-200c low. SESN1 proteins were noticeably reduced in both the PSC and PBC liver specimens in which the expression level of miR-200c was high (Fig. 6C upper). Increased expression of SESN1 proteins was observed in both the PSC and PBC liver specimens in which the expression level of miR-200c was low (Fig. 6C lower). These demonstrated a negative correlation between the expression levels of SESN1 and miR-200c in cholestatic liver diseases. Biliary cellular senescence is an important pathogenic mechanism in PSC^37,38^. The expression level of miR-200c in the PSC livers negatively correlated with that of IL-6 but not CDKN2A, a cell senescence marker, suggesting the possible disassociation between miR-200c expression and biliary senescence (Fig. 6D).

## Discussion

This study is designed to identify the contribution of miRNAs to cholestatic liver diseases. NcRNAs are involved in a wide range of biological functions and processes by regulating diverse modes of molecular feedback and pathways^12,39–41^. MiR-200c is a multi-functional regulator in various biological events, such as epithelial-mesenchymal transition (EMT), apoptosis, and cell senescence^42,43^. During cholestasis, the neuroendocrine-like property and proliferative capacity of cholangiocytes are activated to aggravate the development of biliary liver fibrosis. In this study, we unravel a critical function of miR-200c in governing the homeostasis of cholangiocytes. We demonstrate that miR-200c/SESN1 axis acts upstream of the IL-6/AKT feedback loop to regulate IL-6 production and AKT activation, contributing to cholangiocyte activation and biliary fibrosis (Fig. 6E).

We find that SESN1 is a novel downstream target gene of miR-200c. SESN1 mediates the effect of miR-200c on the proliferative and neuroendocrine-like capacity (of producing the mitogenic IL-6) of cholangiocytes and engages in the pathogenesis of liver fibrosis. SESN1 has been shown to regulate cellular proliferation^44^. Knockdown of SESN1 expression significantly inhibits cell proliferation and accelerates cell senescence in human embryonic fibroblasts^18^. In line with this, we find that SESN1 facilitates proliferation in CA-treated cholangiocytes (Fig. 3E) and in BDL-operated mouse livers (Fig. 4A). To the best of our knowledge, we are the first group to provide evidence for the participation of SESN1 in cholangiocyte proliferation and the pathogenesis of cholestatic liver fibrosis. Although miR-200c regulates expression of SESN1, our correlation analysis suggests that miR-200c might not associate with biliary senescence, opposing to a previous report showing miR-200c induces senescence in human umbilical vein endothelial cells and possibly due to cell specificity^45^.

Our previous study found that hepatic expression of miR-200c was increased in BDL-operated mice, revealed by bulk RNA-sequencing^22^. In the bulk liver, the expression level of miR-200c can be determined by cell types, as well as pathology or disease types (Fig. 6). Moreover, the disease severity may vary a lot individually, thus differentially affect expression of miR-200c. In terms of the individual cell types, expression of miR-200c was elevated moderately in hepatocytes but decreased dramatically in cholangiocytes by BDL (Fig. 1). Cholangiocytes constitute 3~5% of the liver cell population^46^, whereas hepatocytes make up 70–85% of the liver mass^47^. Although the expression level of miR-200c in hepatocytes was much lower than other liver cell types, hepatocytes can be an important source of hepatic miR-200c and miR-200c in hepatocytes may be essential in disease pathogenesis. Indeed, miR-200c-deficiency reduces methionine- and choline-deficient (MCD) diet-induced hepatic steatosis^20^. Tissue-specific *Mir200c*^−/−^ mice are beneficial to further dissect the cell-specific contribution of miR-200c in the pathophysiology of cholestatic and other liver diseases.

Liver fibrosis is characterized by excessive extracellular matrix deposition and HSCs differentiation into myofibroblast-like cells^48^. ACTA2 is one of the most reliable markers of HSC activation^49^. Expression of ACTA2 is markedly increased in *Mir200c*^−/−^-BDL vs. WT-BDL mice but decreased in *Sesn1*^−/−^-BDL vs. *Sesn1*^+/+^-BDL mice (Fig. 4C). These results suggest that miR-200c also regulates HSCs activation in cholestatic liver fibrosis. The role of miR-200c/SESN1 axis will be further elucidated in HSCs.

Hepatic inflammation is an important feature of cholestatic liver diseases in both humans and experimental animals^50^. Studies in BDL-operated mice have demonstrated the crucial role of neutrophils in cholestatic liver injury^51,52^. As demonstrated in this report, the expression level of the neutrophil marker *Ly6g* and the activity of MPO (the most abundantly expressed in neutrophils) are increased in response to BDL, which is further increased in *Mir200c*^−/−^-BDL mice. It is likely that miR-200c also regulates the function of neutrophils during cholestasis, yet expression of miR-200c in neutrophils is unknown. It seems that KCs do not play a role in cholestatic liver injury, as depletion of KCs does not reduce liver injury in BDL-operated mice^53^. Also, there were no differences between WT and *Mir200c*^−/−^ mice after BDL in terms of macrophage marker expression (Fig. 2D).

The PI3K/AKT pathway plays a pivotal role in cholangiocyte proliferation^54^. Activation of AKT is an early event in BAs-mediated cholangiocyte proliferation and secretion^55^. IL-6 triggers the PI3K/AKT pathway that protects against apoptosis and enhances proliferation^56^. AKT increases IL-6 production and thus constitutes a feedforward loop with IL-6^9,10^. Our study finds that the IL-6/AKT loop is activated by BDL, which is boosted by miR-200c-deletion and diminished by SESN1-deletion. This demonstrates that the IL-6/AKT loop can be regulated by miR-200c/SESN1 during cholestasis. Of note, IL-6, along with IL-8 and CCL-5, has been revealed to be a direct target of miR-200c^57^. We do not exclude other mediators downstream of miR-200c in cholestasis and biliary liver fibrosis, but IL-6 can be regarded as a critical contributor to the hepatic damage in miR-200c-deficient BDL mice, given its prominent role in cholangiocyte proliferation and inflammatory responses^4,58^. MiR-200c inhibits the IL-6/AKT loop not only through SESN1-mediated inhibition of AKT but also by directly targeting IL-6. It is possible that AKT is not the only mediator of SESN1 to induce IL-6 expression. Indeed, sestrins also activate Nrf2 to induce IL-6 expression^59^. The molecular mechanism as to how SESN1 activates AKT needs further investigation. As a family of highly conserved stress-inducible proteins, sestrins regulate antioxidant defense and autophagy^60^. In this study, the function of SESN1 is prominent in the presence of cholestasis because SESN1 is induced by BAs in vitro and during cholestasis. Without these, neither cell proliferation nor IL-6 expression is affected by SESN1. In contrast to a previous report that SESN1/2/3 interact and inhibit the activation of JNK in Western diet-induced hepatic lipotoxicity^61^, we find that during cholestasis, SESN1 does not regulate the activity of JNK, ERK, and p38 (Fig. 4D), which is possibly due to distinct etiologies and cell-specific responses.

In summary, our study reveals a novel function of miR-200c as an anti-fibrotic regulator of cholestatic liver fibrosis. In cholestasis, accumulated BAs result in the marked reduction of miR-200c and the subsequent increase of SESN1 in cholangiocytes, which contributes to the activation of the IL-6/AKT loop and increases ductular reaction, biliary hyperplasia, and biliary fibrosis (Fig. 6E). Our findings provide novel mechanistic insights into the role of a miRNA in the pathophysiology of cholestatic liver diseases and suggest that miR-200c may be a new therapeutic target for cholangiopathies.

## Supplementary information

Supporting Information

## Data Availability

The datasets used and/or analyzed during the current study are available from the corresponding author on reasonable request.
