# Supplementary material for: MiR-200c-3p targets SESN1 and represses the IL-6/AKT loop to prevent cholangiocyte activation and cholestatic liver fibrosis
Source: Lab Invest. 2021 Dec 8;102(5):485–93. doi: 10.1038/s41374-021-00710-6 (PMC9042705; doi:10.1038/s41374-021-00710-6)
Supplement: Supplementary file 1 — Supporting Information [file 41374_2021_710_MOESM1_ESM.pdf]

MiR-200c-3p targets SESN1 and represses the IL-6/AKT loop to prevent cholangiocyte activation and cholestatic liver fibrosis

Authors: Yongfeng Song, Melanie Tran, Li Wang, Dong-Ju Shin, and Jianguo Wu

Supporting Information

Contents

Supporting Fig. 1 2
Supporting Fig. 2 3
Supporting Fig. 3 4
Supporting Fig. 4 5
Supporting Fig. 5 6
Supporting Fig. 6 7
Supporting Fig. 7 8
Supporting Fig. 8 9
Supporting Fig. 9 10
Supporting Fig. 10 11
Supporting Fig. 11 12
Supporting Fig. 12 13
Supporting Fig. 13 14
Supporting Fig. 14 15
Supplementary Materials and Methods 16
References 17
Supporting Table 1. Primers used in the study. 18
Supporting Table 2. The information of human liver specimens. 20

Supporting Fig. 1

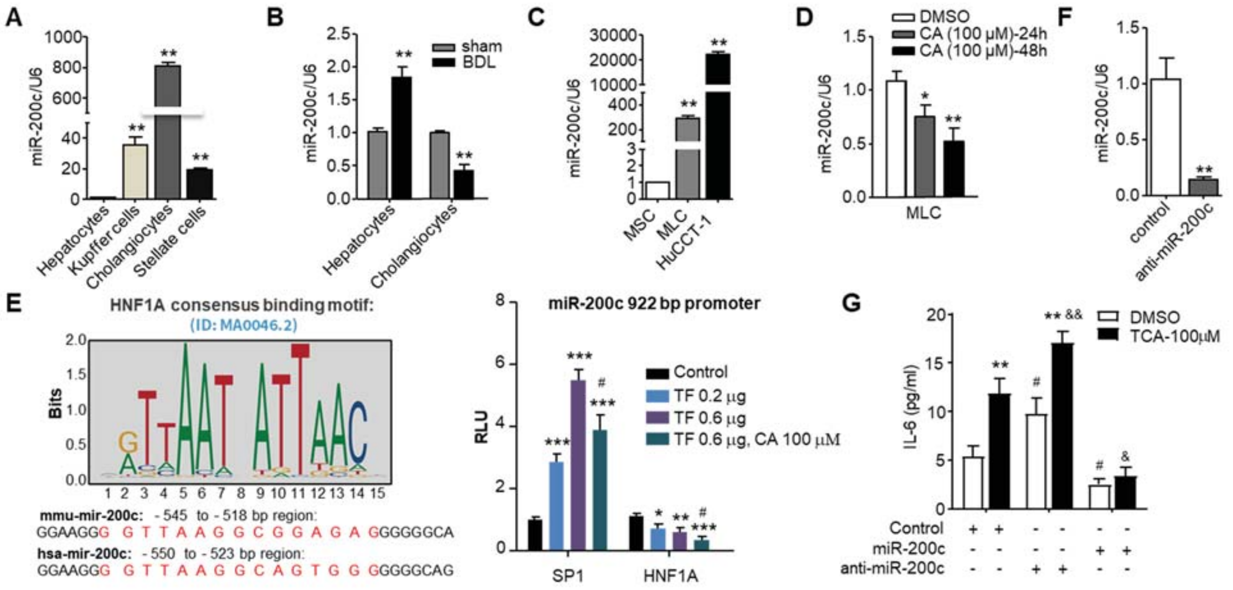

Fig. S1A: qPCR of miR-200c expression levels in primary cells isolated from WT mouse livers. $^{**}P < 0.01$ *vs* hepatocytes.

Fig. S1B: qPCR of miR-200c expression levels in hepatocytes and cholangiocytes isolated from WT-sham and WT-BDL mice. The results are the same as in main Fig. 1A but are plotted as fold changes with sham group set as 1. $^{**}P < 0.01$ *vs* sham.

Fig. S1C: qPCR of miR-200c expression levels in MSC, MLC, and HuCCT-1 cells. $^{**}P < 0.01$ *vs* MSC.

Fig. S1D: qPCR of miR-200c expression levels in MLC cells treated with CA. $^{*}P < 0.05$ & $^{**}P < 0.01$ *vs* DMSO.

Fig. S1E: HNF1A potentially binds and inhibits miR-200c promoter activity. Left: A HNF1A consensus binding motif was retrieved from JASPAR database. The predicted binding sites of HNF1A in the mouse and human miR-200c promoters are in red. Right: Luciferase assay showing that HNF1A inhibited miR-200c promoter activity in MLC cells without or with CA treatment. Data are shown as mean ± SEM (triplicate assays). Specificity protein 1 (SP1) was used as a positive control. TF, transcription factor SP1 or HNF1A. $^{*}P < 0.05$, $^{**}P < 0.01$ & $^{***}P < 0.001$ *vs* control; $^{\#}P < 0.05$ *vs* TF 0.6 μg.

Fig. S1F: qPCR of miR-200c expression levels in MLC cells transfected with control or miR-200c inhibitor (anti-miR-200c) plasmid for 24 hrs. $^{**}P < 0.01$ *vs* control.

Fig. S1G: ELISA of IL-6 secretion from MLC cells which were transfected with plasmids of control miRNA, miR-200c, or anti-miR-200c, and then treated with DMSO or TCA (100 μM) for 48 hrs. Data are shown as mean ± SEM (triplicate assays). $^{**}P < 0.01$ TCA-100 μM *vs* DMSO; $^{\#\#}P < 0.01$ *vs* control-DMSO; $^{\&}P < 0.05$ & $^{\&\&}P < 0.01$ *vs* control-TCA-100 μM.

Supporting Fig. 2

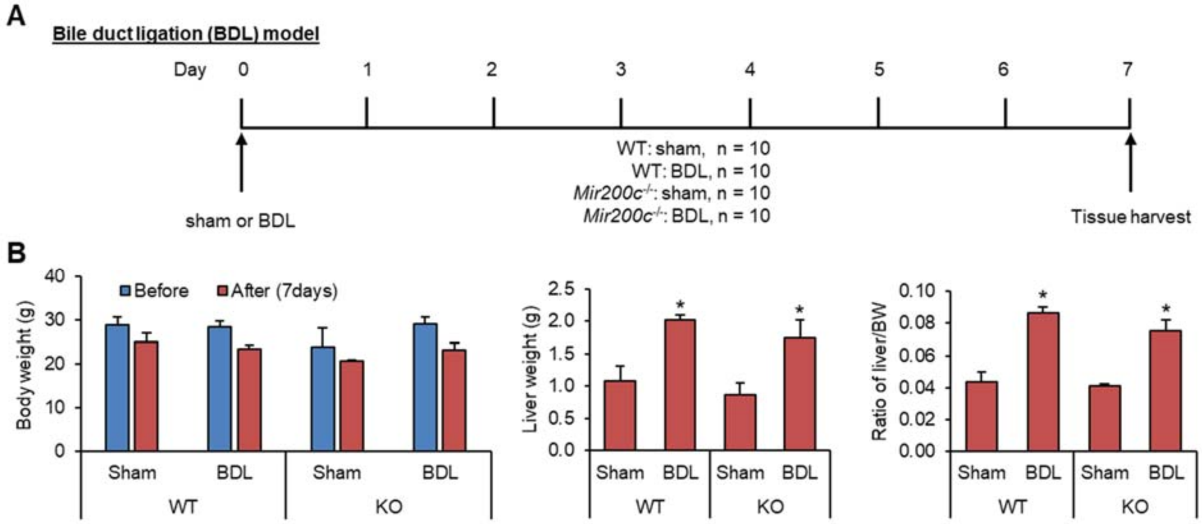

Fig. S2A: The diagram showing the mouse BDL model used in this study. Considering that some mice may not survive from BDL surgery for various reasons, we used 10 mice/group.

Fig. S2B: The body and liver weight of *Mir200c*$^{+/+}$ (WT) and *Mir200c*$^{-/-}$ (KO) mice before and after BDL. Data are shown as mean ± SEM. *P < 0.05 *vs* sham.

Supporting Fig. 3

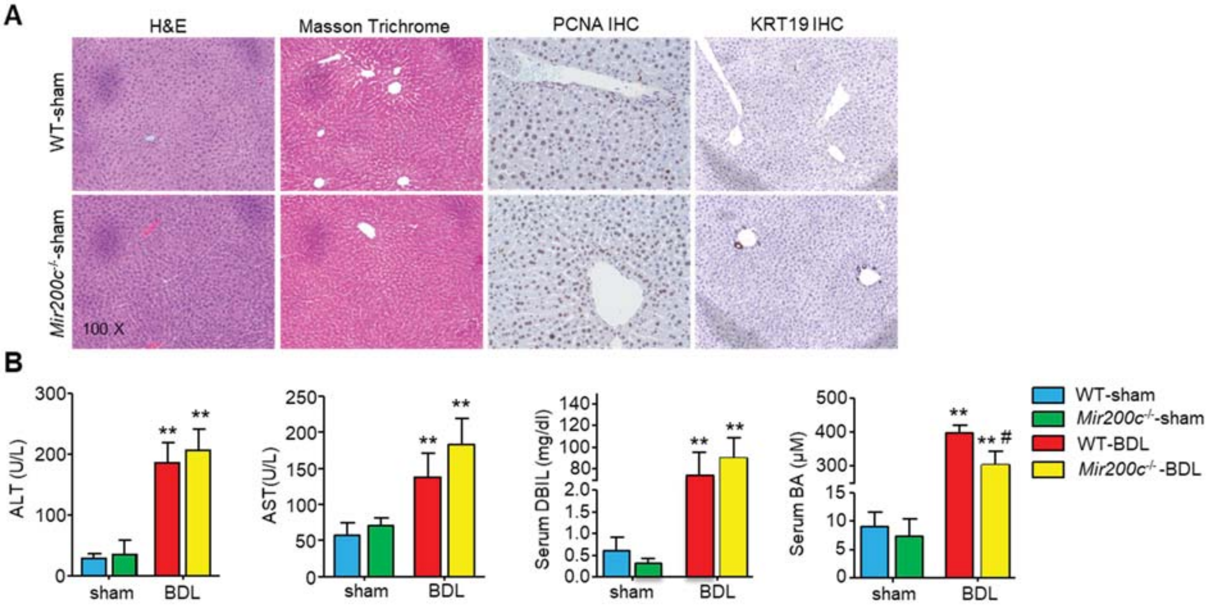

Fig. S3A: Representative images of H&E, Masson Trichrome, and IHC of PCNA and KRT19 staining of liver sections in the sham groups of WT and *Mir200c*$^{-/-}$ mice.

Fig. S3B: Serum ALT, AST, DBIL, and BA levels in WT and *Mir200c*$^{-/-}$ mice after sham or BDL surgery. Data are shown as mean ± SEM (n = 10/group). $^{**}P < 0.01$ *vs* sham; $^{\#}P < 0.05$ *vs* WT-BDL.

Supporting Fig. 4

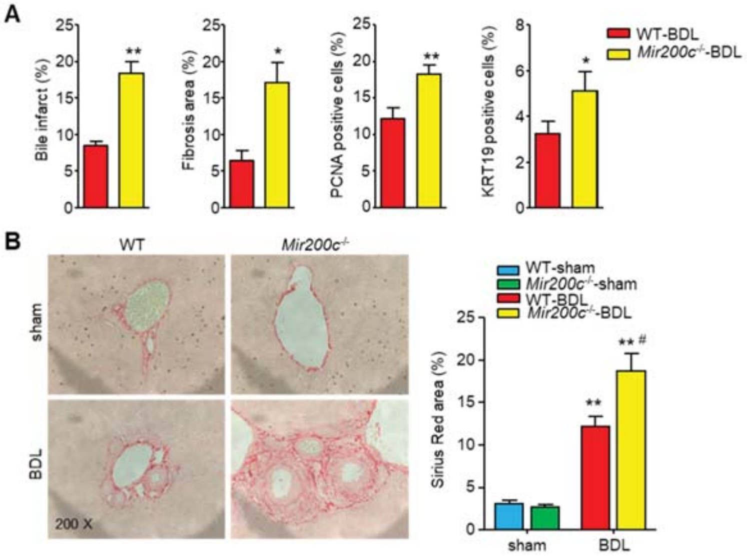

Fig. S4A: Quantification of the bile infarct area in BDL-mice and the results in main Fig. 2A. Five fields of view per slide per mouse liver were randomly taken under a microscope and quantified by ImageJ. n = 10 mice/group. **P* < 0.05 & ***P* < 0.01 *vs* WT-BDL.

Fig. S4B: Representative images of Sirius Red staining of liver sections from WT and *Mir200c*$^{-/-}$ mice under sham or BDL surgery. The quantification was the same as Fig. S4A. n = 10 mice/group. ***P* < 0.01 *vs* sham; $^{\#}$*P* < 0.05 *vs* WT-BDL.

Supporting Fig. 5

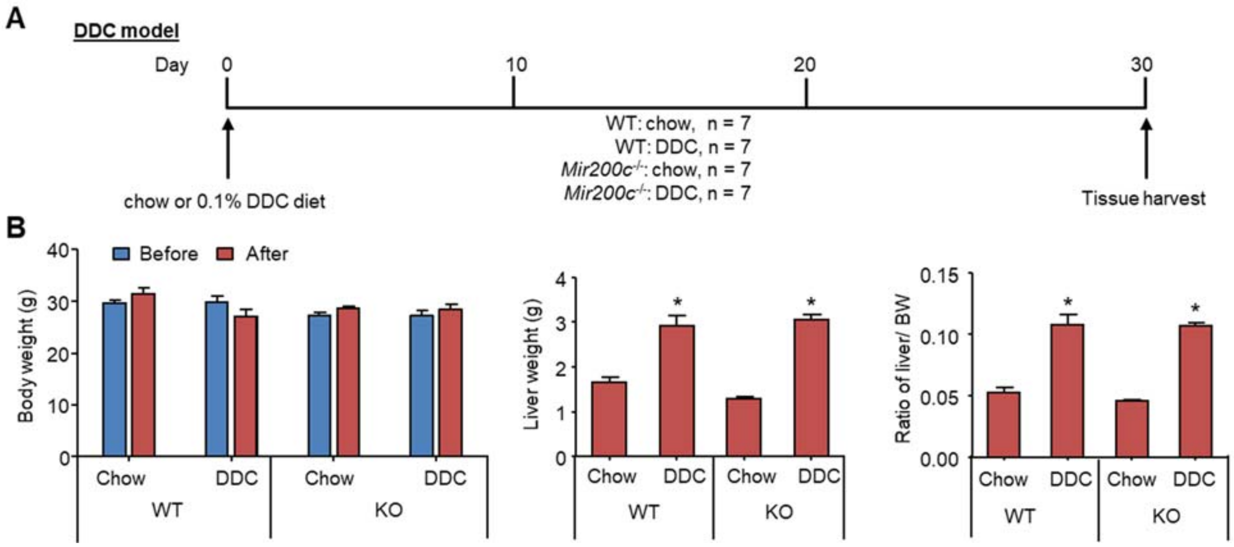

Fig. S5A: The diagram showing the DDC-feeding model used in this study.

Fig. S5B: The body and liver weight of *Mir200c*$^{+/+}$ (WT) and *Mir200c*$^{-/-}$ (KO) mice before and after DDC feeding. Data are shown as mean ± SEM. *$P < 0.05$ *vs* chow.

Supporting Fig. 6

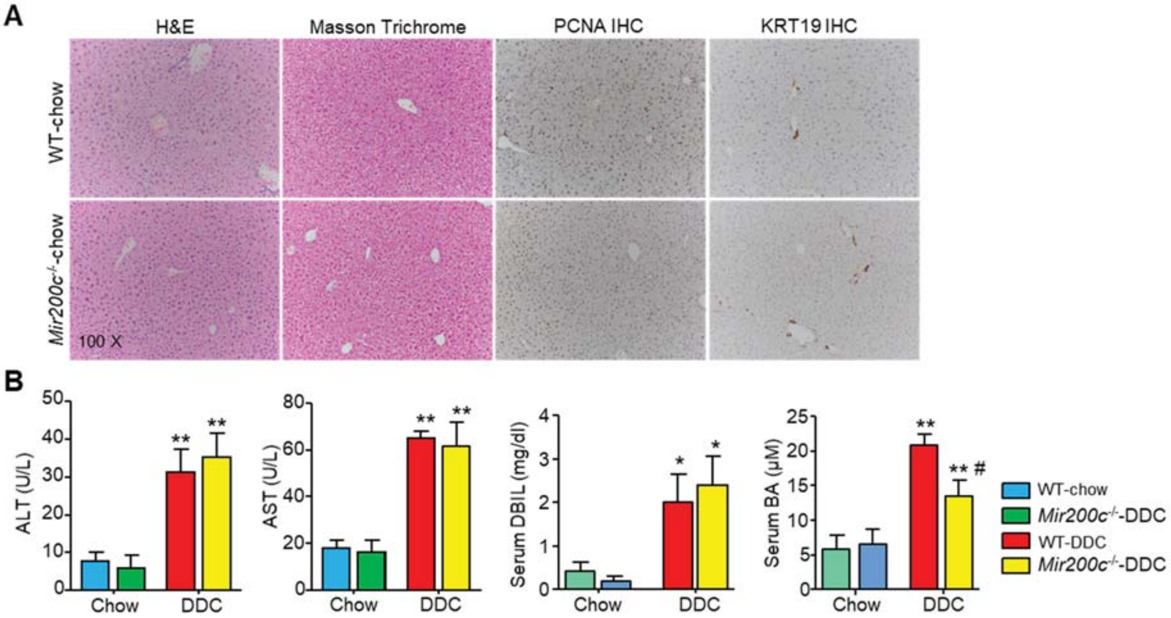

Fig. S6A: Representative images of H&E, Masson Trichrome, and IHC of PCNA and KRT19 IHC staining of liver sections in control chow groups of WT and *Mir200c*$^{-/-}$ mice.

Fig. S6B: Serum ALT, AST, DBIL, and BA levels in chow and DDC groups of WT and *Mir200c*$^{-/-}$ mice. Data are shown as mean ± SEM (n = 7/group). **P* < 0.05 & ***P* < 0.01 *vs* chow; $^{\#}$*P* < 0.05 *vs* WT-DDC.

Supporting Fig. 7

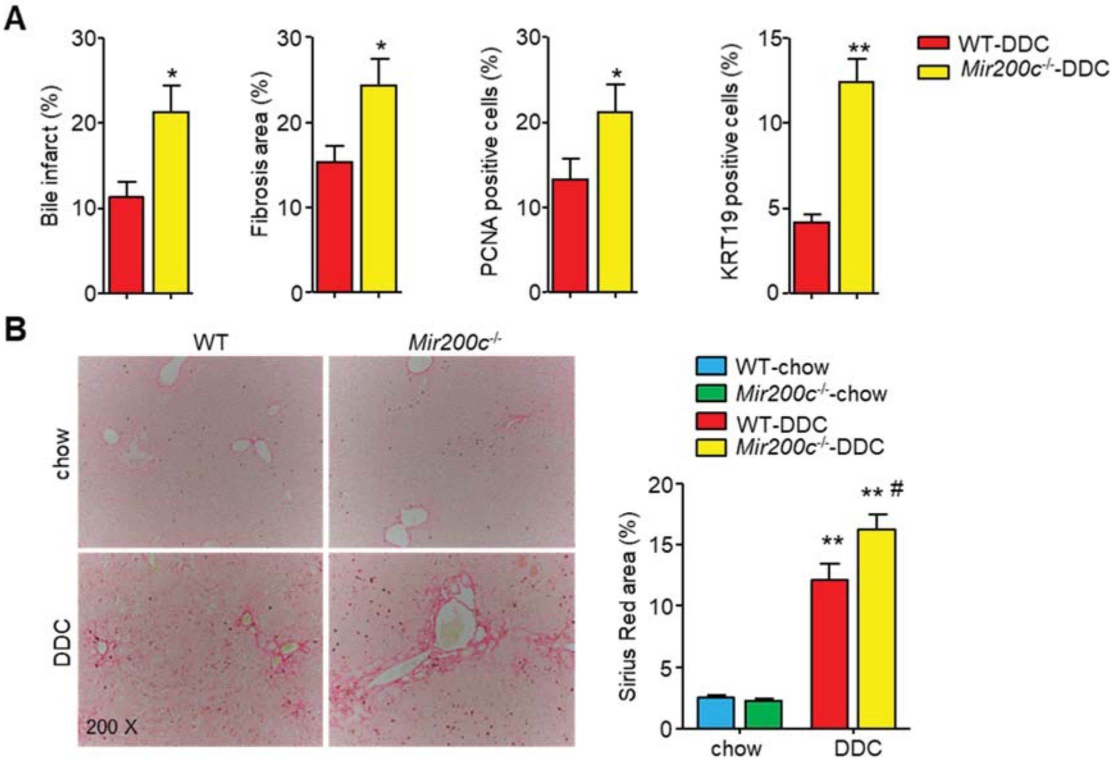

Fig. S7A: Quantification of the bile infarct area in DDC-fed mice and the results in main Fig. 2B. Five fields of view per slide per mouse liver were randomly taken under a microscope and quantified by ImageJ. n = 7 mice/group. **P* < 0.05 & ***P* < 0.01 *vs* WT-DDC.

Fig. S7B: Representative images of Sirius Red staining of liver sections of WT and *Mir200c*$^{-/-}$ mice fed a chow or DDC diet for 1 month. The quantification was the same as Fig. S7A. n = 7 mice/group. ***P* < 0.01 *vs* chow; $^{\#}$*P* < 0.05 *vs* WT-DDC.

Supporting Fig. 8

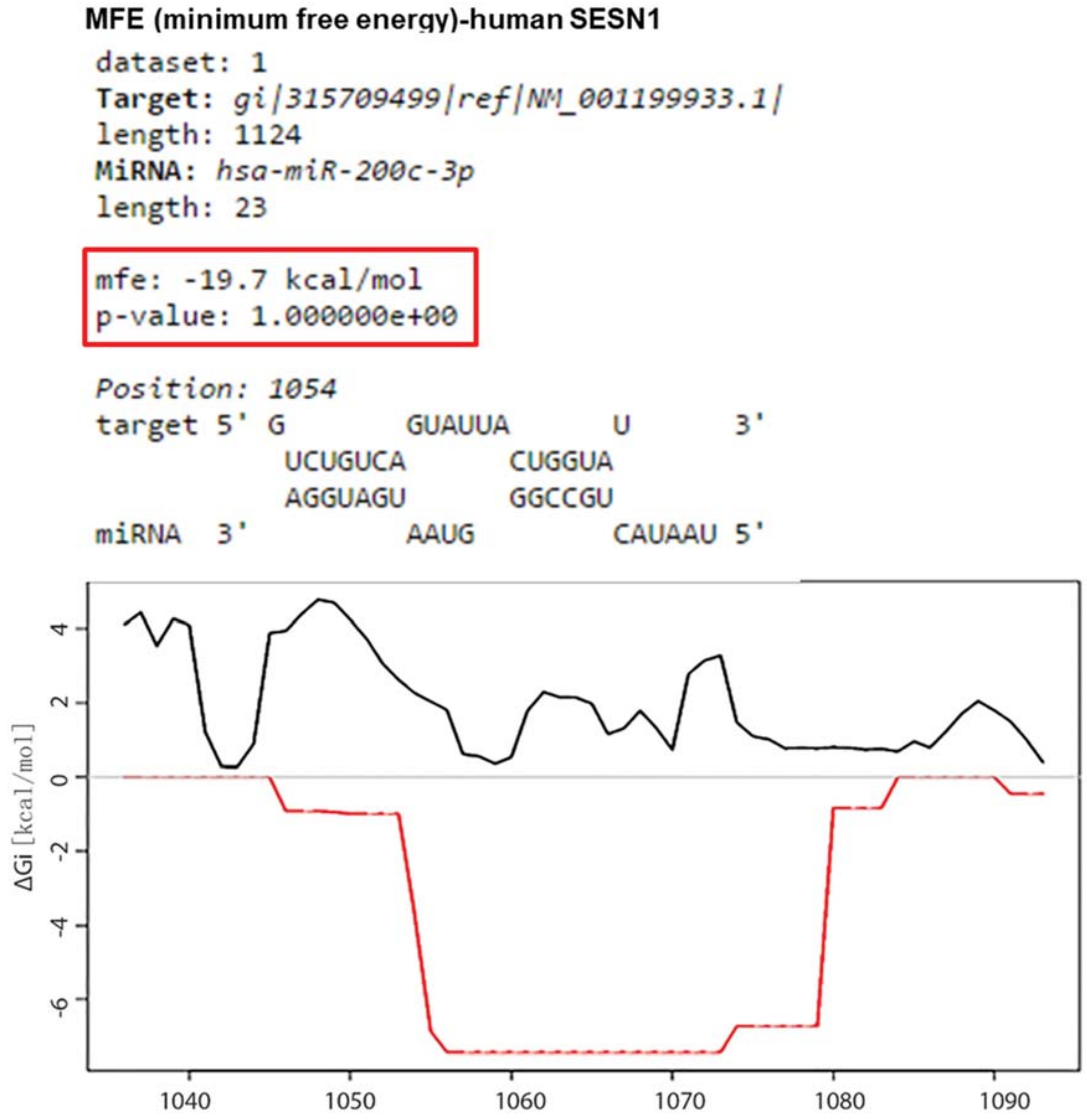

Fig. S8: The free energy value of miR-200c binding to human SESN1 3′-UTR.

Supporting Fig. 9

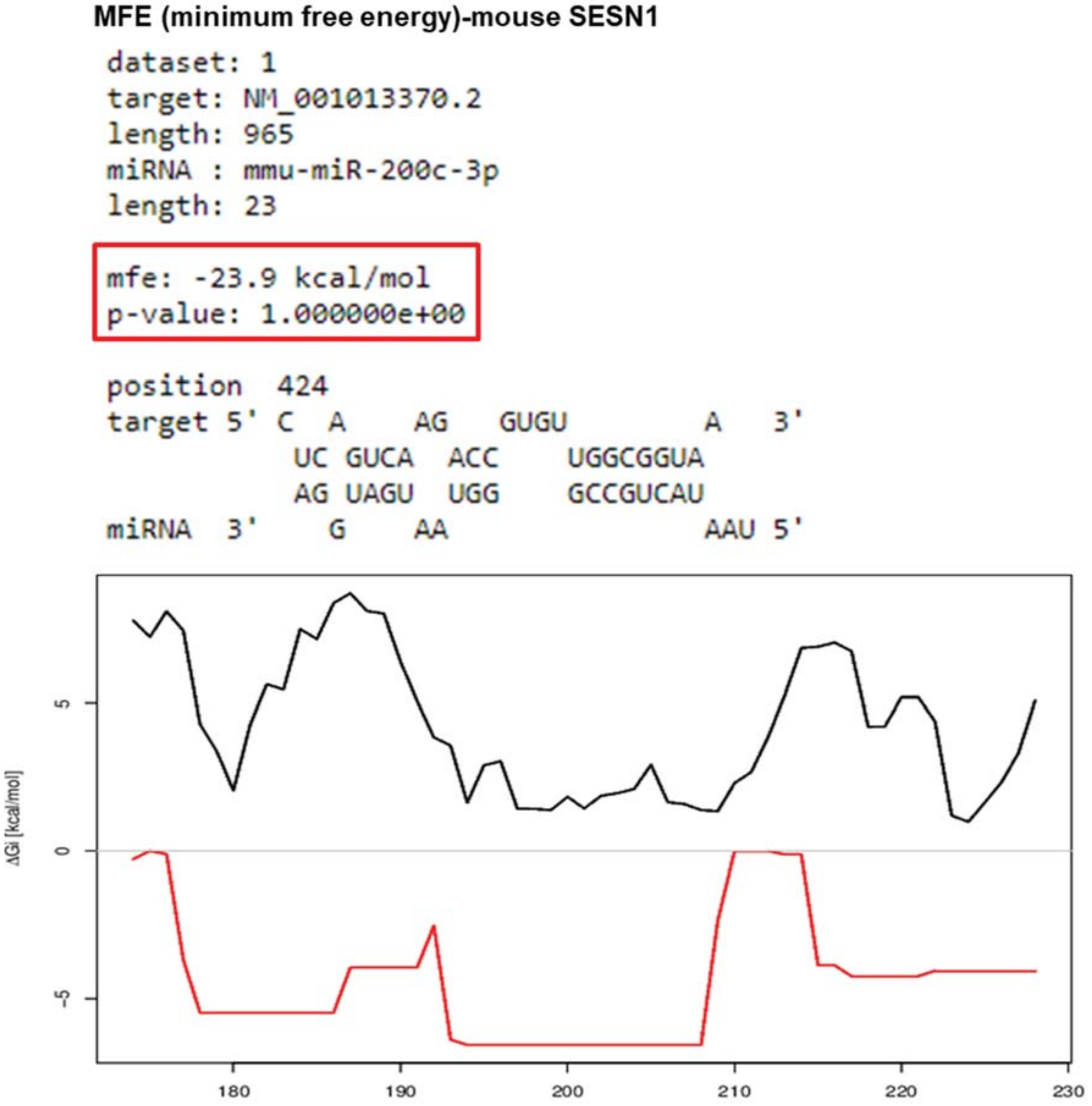

Fig. S9: The free energy value of miR-200c binding to mouse SESN1 3′-UTR.

Supporting Fig. 10

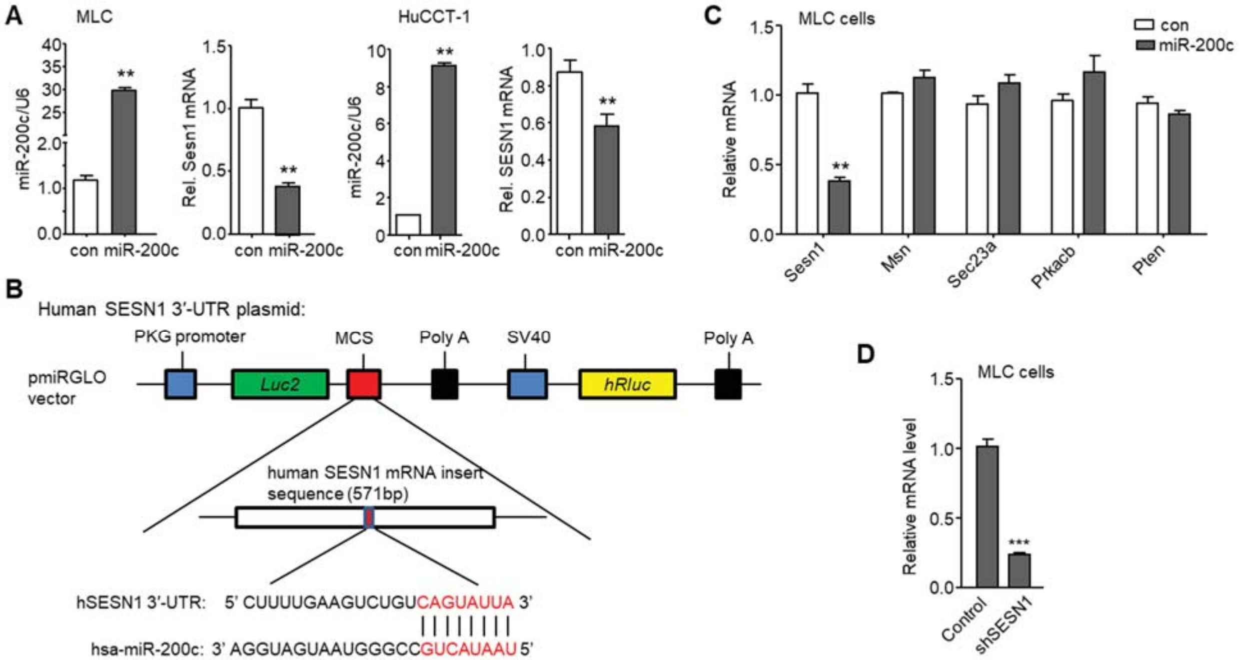

Fig. S10A: qPCR of miR-200c and SESN1 mRNA levels in MLC and HuCCT-1 cells transfected with control (con) or miR-200c expression plasmids. $^{**}P < 0.01$ *vs* con.
Fig. S10B: The diagram showing the cloning of the human SESN1 (hSESN1) 3′-UTR luciferase reporter.
Fig. S10C: qPCR of mRNA levels of the predicted miR-200c target genes in MLC cells transfected with control (con) or miR-200c expression plasmids. $^{**}P < 0.01$ *vs* con.
Fig. S10D: qPCR of SESN1 mRNA levels in MLC cells transfected with control or SESN1 shRNAs. $^{***}P < 0.001$ *vs* control.

Supporting Fig. 11

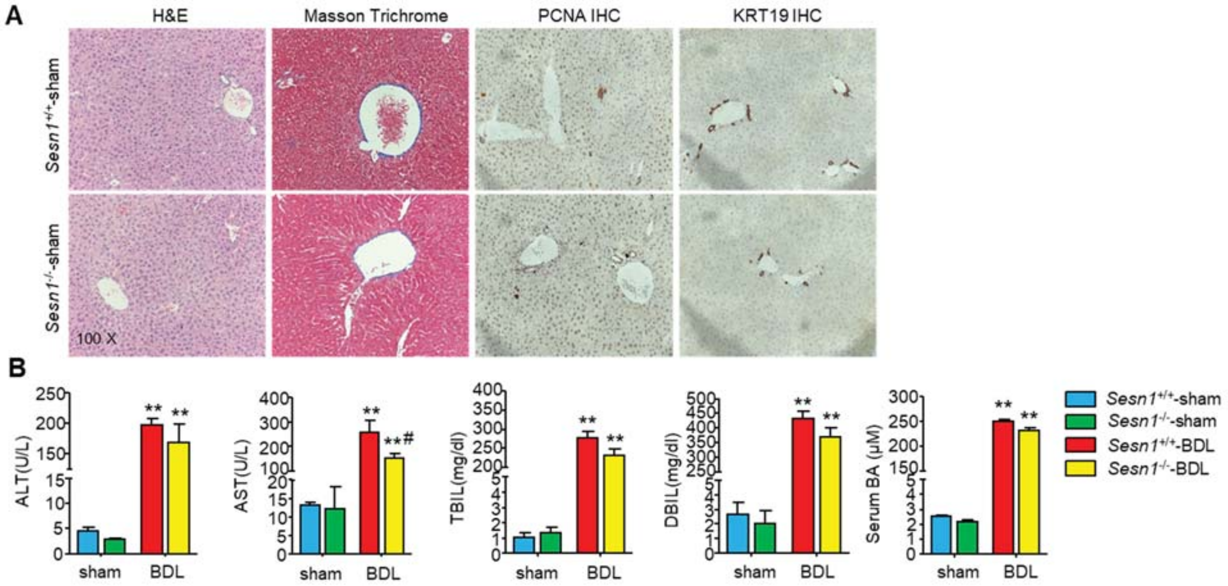

Fig. S11A: Representative images of H&E, Masson Trichrome, and IHC of PCNA and KRT19 staining of liver sections in sham groups of *Sesn1*$^{+/+}$ and *Sesn1*$^{-/-}$ mice.

Fig. S11B: Serum ALT, AST, TBIL, DBIL, and BA levels in sham and BDL groups of *Sesn1*$^{+/+}$ and *Sesn1*$^{-/-}$ mice. Data are shown as mean ± SEM (n=10/group). $^{**}P < 0.01$ *vs* sham; $^{\#}P < 0.05$ *vs* *Sesn1*$^{+/+}$-BDL.

Supporting Fig. 12

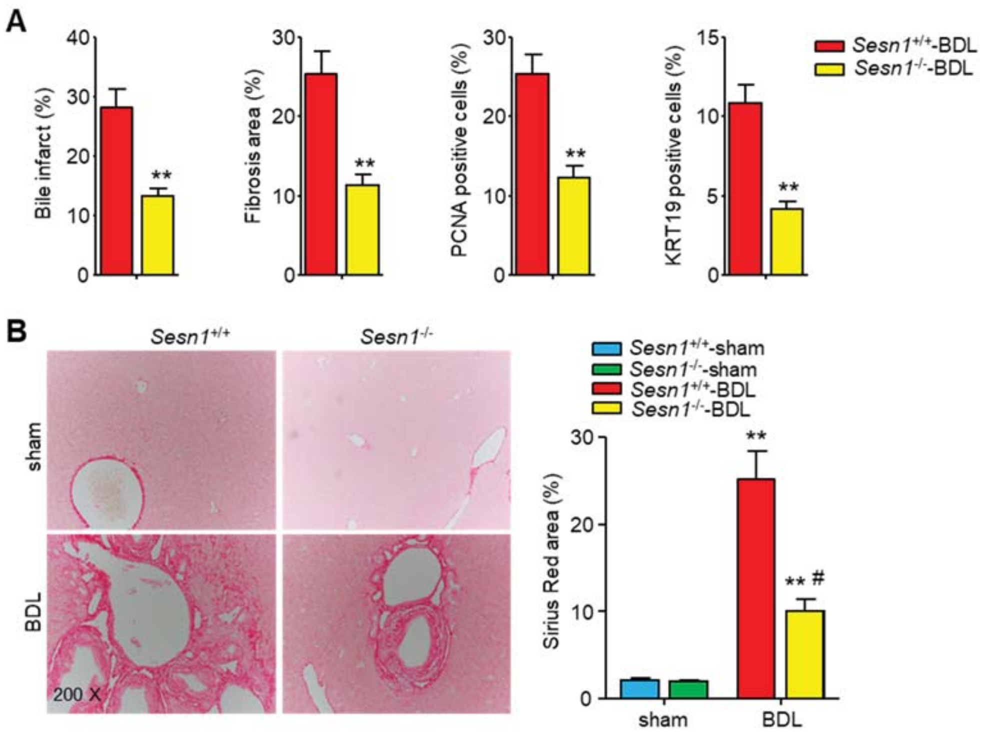

Fig. S12A: Quantification of the bile infarct area in BDL-mice and the results in main Fig. 4A. Five fields of view per slide per mouse liver were randomly taken under a microscope and quantified by ImageJ. n = 10 mice/group. $^{**}P < 0.01$ *vs* *Sesn1*$^{+/+}$-BDL.

Fig. S12B: Representative images of Sirius Red staining of liver sections from *Sesn1*$^{+/+}$ and *Sesn1*$^{-/-}$ mice under sham or BDL surgery. The quantification was the same as Fig. S12A. n = 10 mice/group. $^{**}P < 0.01$ *vs* sham; $^{\#}P < 0.05$ *vs* *Sesn1*$^{+/+}$-BDL.

Supporting Fig. 13

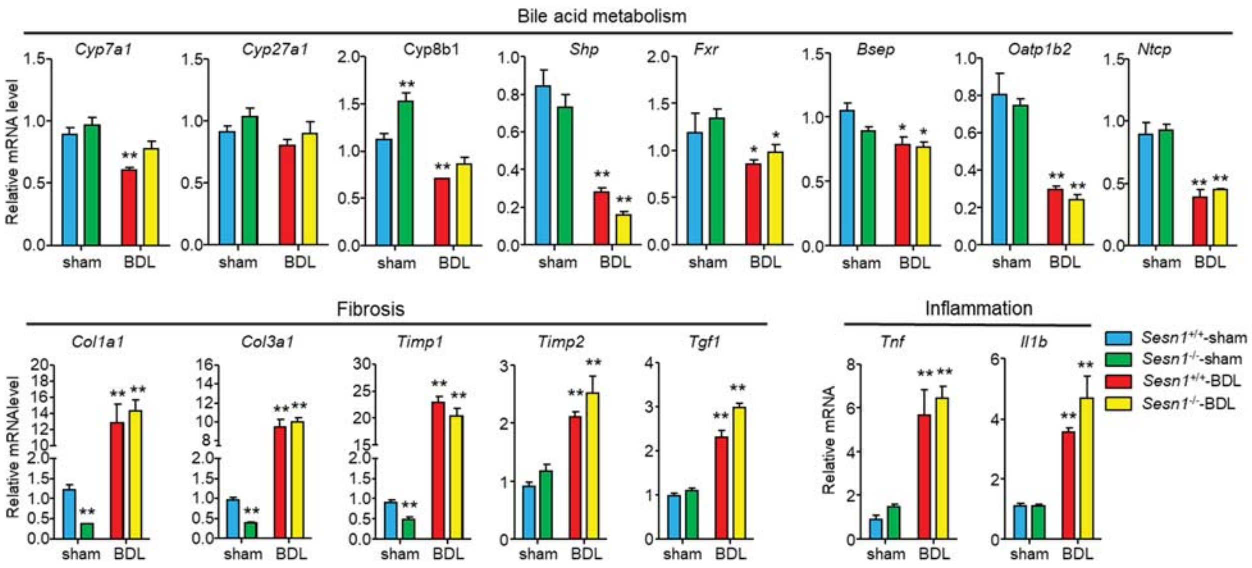

Fig. S13: qPCR of gene expression in several pathways in *Sesn1*$^{+/+}$ and *Sesn1*$^{-/-}$ mice under sham or BDL surgery for one week. Data are shown as mean ± SEM (n = 10/group). **P* < 0.05 & ***P* < 0.01 *vs* sham.

Supporting Fig. 14

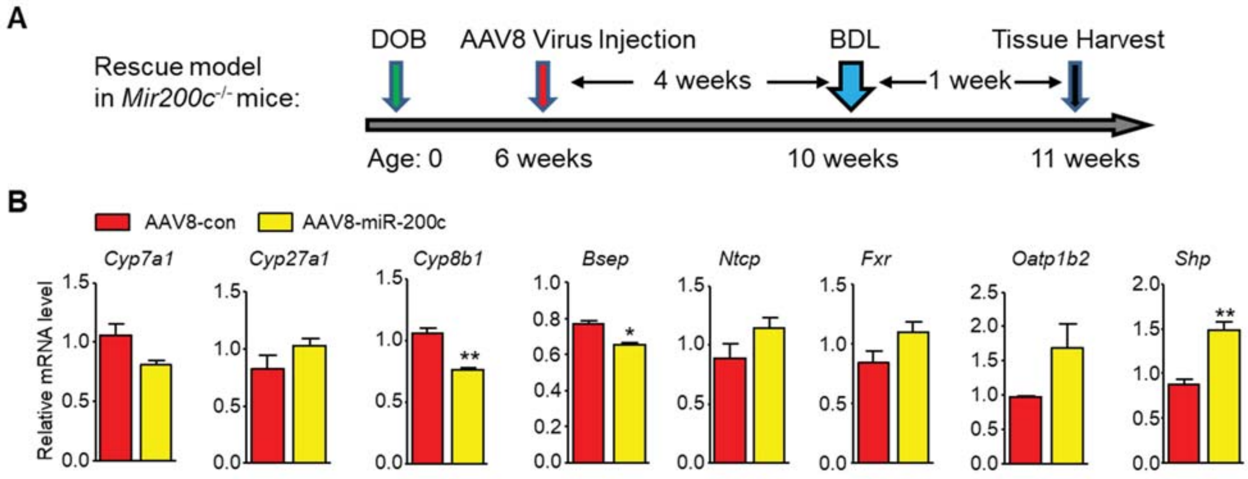

Fig. S14A: The diagram showing the mouse model used for miR-200c rescue experiment. *Mir200c*$^{-/-}$ mice were transduced with AAV8-miR-200c or AAV8-con (control) for 1 month via tail vein-injection followed by BDL for 7 days.

Fig. S14B: qPCR of gene expression in several pathways in mouse livers from the rescue experiment. Data are shown as mean ± SEM (n = 5 mice/group). **P* < 0.05 & ***P* < 0.01 *vs* AAV8-con.

Supplementary Materials and Methods

Human liver specimens

The coded human liver specimens of normal controls, primary sclerosing cholangitis (PSC), primary biliary cholangitis (PBC), and congenital hepatic fibrosis (CHF) were obtained through the Liver Tissue Cell Distribution System (Minneapolis, Minnesota) which was funded by NIH (Contract # HSN276201200017C). Because we did not ascertain individual identities associated with the samples, the Institutional Review Board for Human Research at the University of Connecticut determined that the project was not research involving human subjects. The basic demographic characteristics are listed in Supporting Table 2.

Cell lines, plasmids, and antibodies

Culture conditions of mouse small cholangiocytes (MSC), mouse large cholangiocytes (MLC), human cholangiocarcinoma cell line (HuCCT-1), and human embryonic kidney 293T cells were described previously [1, 2]. MiR-200c overexpression plasmid was obtained from Cell Biolabs. SP1 (#12097) and HNF1A (#31104) plasmids were originally from Addgene. SESN1 3'UTR was cloned into pmirGlo (Promega) to obtain a luciferase reporter construct. The SESN1 3'UTR mutant plasmid was constructed using Q5 Site-Directed Mutagenesis Kit (NEB). The luciferase reporters containing upstream fragments of miR-200c (- 3 kb and - 922 bp) were described previously [3]. SESN1 shRNA (TRCN0000087812) was from MilliporeSigma. Antibodies were purchased from Sigma-Aldrich (α-SMA), Cell Signaling (p-AKT, AKT, p-ERK1/2, ERK1/2, p-JNK1/2, JNK1/2, β-actin), GeneTex (SESN1), and Santa Cruz (α-tubulin) [2, 4].

BrdU incorporation

Cell proliferation was measured using the BrdU-ELISA kit purchased from MilliporeSigma. In brief, MLC cells were seeded at a 50-60% confluency into 96-well plates and transfected with plasmids. After 24 hr, cells were washed with starvation medium and incubated with either

growth medium (positive control), starvation medium (background control and negative control), or starvation medium supplemented with CA for 24 hr. Subsequently, fresh medium containing bromodeoxyuridine (BrdU) was added to wells except the background control. After 24 hr, the BrdU-ELISA was carried out according to the manufacturer's protocol.

References

1 Song Y, Liu C, Liu X, Trottier J, Beaudoin M, Zhang L, et al. H19 promotes cholestatic liver fibrosis by preventing ZEB1-mediated inhibition of epithelial cell adhesion molecule. *Hepatology* **66**, 1183-1196 (2017).

2 Wu J, Zhao Y, Park YK, Lee JY, Gao L, Zhao J, et al. Loss of PDK4 switches the hepatic NF-kappaB/TNF pathway from pro-survival to pro-apoptosis. *Hepatology* **68**, 1111-1124 (2018).

3 Zhang Y, Yang Z, Whitby R, Wang L. Regulation of miR-200c by nuclear receptors PPARalpha, LRH-1 and SHP. *Biochem Biophys Res Commun* **416**, 135-139 (2011).

4 Tran M, Lee SM, Shin DJ, Wang L. Loss of miR-141/200c ameliorates hepatic steatosis and inflammation by reprogramming multiple signaling pathways in NASH. *JCI Insight* **2**, e96094 (2017).

Supporting Table 1. Primers used in this study.

Name	Forward	Reverse
**qPCR primers**		
miR-200c	GTGCAGGGTCCGAGGT	CGCTAATACTGCCGGGTAATG
miR-141	GTGCAGGGTCCGAGGT	GCCCGCTAACACTGTCTGGTAAAG
Mouse Acta2 (a-SMA)	AGAGTTACGAGTTGCCTGATG	ATGAAGGATGGCTGGAACAG
Mouse Timp1	ATTCAAGGCTGTGGGAAATG	CTCAGAGTACGCCAGGGAAC
Mouse Timp2	TCCTTGCTACAGGCAGGAGT	CATTCGCTGAAGTCTGTGGA
Mouse col1a1	GACGCCATCAAGGTCTACTGC	GGAAGGTCAGCTGGATAGCG
Mouse col1a2	AAGGATACAGTGGATTGCAGG	TCTACCATCTTTGCCAACGG
Mouse Col3a1	TGGTCCTCAGGGTGTAAAGG	GTCCAGCATCACCTTTTGGT
Mouse Il6	CATGTTCTCTGGGAAATCGTGG	GTACTCCAGGTAGCTATGGTAC
Mouse Il1b	TGCCACCTTTTGACAGTGATG	GCTGCGAGATTTGAAGCTGG
Mouse Nlrp3	ATGGTATGCCAGGAGGACAG	ATGCTCCTTGACCAGTTGGA
Mouse Tnf	ACGGCATGGATCTCAAAGAC	CGGACTCCGCAAAGTCTAAG
Mouse Krt7 (Ck7)	TGAGATTGCGGAGATGAACC	CGATGCTGGACTCTAACTTGG
Mouse Krt19 (Ck19)	CTCCCGAGATTACAACCACTAC	GTTCTGTCTCAAACTTGGTTCTG
Mouse Icam1	AGATCACATTCACGGTGCTGGCTA	AGCTTTGGGATGGTAGCTGGAAGA
Mouse Vcam1	CTGGGGCAGGAAGTTAGATA	CAAGAAAAAGAAGGGGAGTCA
Mouse Ccl2 (Mcp1)	AGCAGGTGTCCCAAAGAAGCTGTA	AAAGGTGCTGAAGACCTTAGGGCA
Mouse Ly6g	GAGAGGAAGTTTTATCTGTGCAGC	TCTCAGGTGGGACCCCAATA
Mouse Ccl3 (Mip1a)	ATTCCACGCCAATTCATCGT	CAGGTCTCTTTGGAGTCAGC
Mouse Ccl4 (Mip1b)	TATGAGACCAGCAGTCTTTGC	TCAACTCCAAGTCACTCATGT
Mouse Sox9	CAAGACTCTGGGCAAGCTC	GGGCTGGTACTTGTAATCGG
Mouse Pcna	TTTGAGGCACGCCTGATCC	GGAGACGTGAGACGAGTCCAT
Mouse Tgf1	GTCAGACATTCGGGAAGCAG	GCGTATCAGTGGGGGTCA
Mouse Adgre1 (F4/80)	TCAAATGGATCCAGAAGGCTCCCA	TGCACTGCTTGGCATTGCTGTATC
Mouse Cd68	GCTGTTCACCTTGACCTGCTC	GGTTGATTGTCGTCTGCGG
Mouse Oatp1b2	GGGAACATGCTTCGTGGGATA	GCAGTTATGCGGACACTTCTC
Mouse Bsep	ATGTGACCTTCCATTATCCTTCTCG	GCTGTAGTGCTGTGCTCTTCC

Mouse Ntcp	TCATCTGCGGCTGCTCTCC	TGGTCATCACAATGCTGAGGTTC
Mouse Sesn1	CTGCTTTGGGTCGTCTGGAT	CTGCCGCCATGATTCCAATG
Mouse Msn	GCAGAATGACAGACTGACTCC	CGCTTGTTAATCCGAAGCCG
Mouse Sec23a	GGCAGATGCTCAAACTCAAATC	GAGTTGTCTGTCCAACCATCTTA
Mouse Prkacb	TTCGGGTTCGCCAAAAGAGT	CCAGTCCACCGCCTTATTGT
Mouse Pten	CCAGACATGACAGCCATCATCA	CTGCAGGAAATCCCATAGCAATA
Mouse Cyp7a1	GGGATTGCTGTGGTAGTGAGC	GGTTATGGAATCAACCCGTTGT
Mouse Cyp27a1	GGAGCCACGACCCTAGATG	GCCATGCCAAGATAAGGAAGC
Mouse Fxr	GCTTGATGTGCTACAAAAGCT	CGTGGTGATGGTTGAATGTCC
Mouse Cyp8b1	CCTCTGGACAAGGGTTTTGTG	GCACCGTGAAGACATCCCC
Mouse Shp	CAGGTCGTCCGACTATTCTG	ACTTCACACAGTGCCCAGTG
Human CDKN2A	CCAACGCACCGAATAGTTACG	GCGCTGCCCATCATCATG
**Loading control**		
Mouse Hprt	CGTCGTGATTAGCGATGATGA	CACACAGAGGGCCACAATGT
Mouse/human U6	CTCGCTTCGGCAGCACA	AACGCTTCACGAATTTGCGT
Human GAPDH	GAAATCCCATCACCATCTTCCA	CAGCATCGCCCCACTTG
**Cloning construct primers**		
SESN1 3'-UTR plasmid	CGCGAGCTCttaaacttgcattatgtgtt	CCCTCGAGctttatttaaaaataccagt
SESN1 3'-UTR mutant	AATACCAGTAcgcCTGACAGACTTCAAAAG CAATTC	TTTAAATAAAGCTCGAGTCTAG
**Stem-loop primer for miRNA reverse transcription**		
miR-200c	GTCGTATCCAGTGCAGGGTCCGAGGTATTCGCACTGGATACGACCCATCA	

Supporting Table 2. The information of human liver specimens.

No.	Disease	Age (year)	Gender	Individual ID
1	Normal	47	F	HH-1220
2	Normal	58	F	HH-1223
3	Normal	56	M	HH-1234
4	Normal	55	F	HH-1274
5	Normal	36	F	HH-1280
6	Normal	70	M	HH-1246
7	Normal	59	M	HH-1249
8	Normal	59	M	HH-1278
9	Normal	50	M	HH-1287
10	Normal	67	F	HH-1320
1	PSC	32	F	UMN-1163
2	PSC	57	F	UMN-1171
3	PSC	34	F	UMN-1178
4	PSC	22	F	UMN-1210
5	PSC	51	F	UMN-1240
6	PSC	27	F	UMN-1358
7	PSC	53	F	UMN-1635
8	PSC	63	F	UMN-1690
9	PSC	41	M	UMN-1150
10	PSC	51	M	UMN-1155
11	PSC	52	M	UMN-1179
12	PSC	55	M	UMN-1182
13	PSC	25	M	UMN-1199
14	PSC	41	M	UMN-1222
15	PSC	50	M	UMN-1236
16	PSC	38	M	UMN-1267
17	PSC	62	M	UMN-1270
18	PSC	32	M	UMN-1307
19	PSC	38	M	UMN-1315
20	PSC	58	M	UMN-1339
21	PSC	59	M	UMN-1363
22	PSC	48	M	UMN-1383
23	PSC	53	M	UMN-1465
24	PSC	58	M	UMN-1489
25	PSC	47	M	UMN-1520
26	PSC	61	M	UMN-1534
27	PSC	43	M	UMN-1560
28	PSC	60	M	UMN-1600
29	PSC	33	M	UMN-1614
30	PSC	51	M	UMN-1645

31	PSC	58	M	UMN-1518
1	PBC	58	F	UMN-1006
2	PBC	63	F	UMN-1038
3	PBC	62	F	UMN-1054
4	PBC	55	F	UMN-1064
5	PBC	60	F	UMN-1173
6	PBC	59	F	UMN-1183
7	PBC	46	F	UMN-1213
8	PBC	59	F	UMN-1218
9	PBC	46	F	UMN-1234
10	PBC	52	F	UMN-1269
11	PBC	48	F	UMN-1374
12	PBC	64	F	UMN-1448
13	PBC	65	F	UMN-1490
14	PBC	60	F	UMN-1595
15	PBC	63	F	UMN-1682
16	PBC	62	M	UMN-1003
17	PBC	63	M	UMN-1292
18	PBC	35	M	UMN-1378
19	PBC	65	M	UMN-1388
20	PBC	67	M	UMN-1441
21	PBC	52	M	UMN-1679
1	CHF	13.14	M	UMN-916
2	CHF	13.1	M	UMN-1025
3	CHF	7.42	M	UMN-1028
4	CHF	1.79	F	UMN-1120
5	CHF	unknown	unknown	UMN-742
